# Dietary Vitamin Intakes, Reference-Value Profiles, and Major Food Sources in a Historical Cohort of Geographically Diverse UK University Students

**DOI:** 10.3390/foods15213997

**Published:** 2026-11-07

**Authors:** Antonio Peña-Fernández, Guillermo Torrado Durán, M. Ángeles Peña Fernández

**Affiliations:** 1 Area of Legal and Forensic Medicine, Department of Surgery, Medical and Social Sciences, Faculty of Medicine and Health Sciences, University of Alcalá, Ctra. Madrid-Barcelona, Km. 33.6, 28871 Alcalá de Henares, Spain; 2 Leicester School of Allied Health Sciences, De Montfort University, Leicester LE1 9BH, UK; 3 Department of Biomedical Sciences, Faculty of Pharmacy, University of Alcalá, Crta. Madrid-Barcelona Km. 33.6, 28871 Alcalá de Henares, Spain

**Keywords:** vitamins, young adults, university students, vitamin D, folate, dietary reference values, food sources, Nutritics, CoFID, United Kingdom

## Abstract

Understanding the foods that supply vitamins can inform dietary strategies for young adults. This historical cross-sectional study assessed intakes and food-group sources of 11 vitamins in 111 students aged 18–22 years at an English university in 2016. Dietary intake was estimated using an adapted food-frequency questionnaire and Nutritics. Food source contributions were independently reconstructed using UK food-composition data and calibrated against Nutritics totals. Fish and seafood supplied 31.8% of vitamin D; red and processed meat, dairy and alternatives, and fish and seafood together supplied 79.2% of vitamin B12. Fruit and vegetables contributed 86.3% of vitamin C, whereas vegetables (31.0%), cereals and grains (18.3%), and fruit (16.7%) were the principal CoFID-based total-folate sources. Vitamin K1 source reconstruction remained exploratory owing to incomplete coverage. Dietary vitamin D intake was below the UK reference nutrient intake in 94.6% of participants; 27.9% and 48.6% were below the European folate average requirement and population reference intake, respectively. Conversely, 93.7% did not fall below any of the seven UK lower reference nutrient intakes. Adjusted vitamin A intake was lower among males (*q* = 0.029), and several vitamins differed by geographical background. These findings demonstrate the value of combining food source attribution with nutrient-specific reference comparisons to guide food-based nutritional research.

## 1. Introduction

Emerging adulthood represents a distinct life-course period during which increasing autonomy over food purchasing, meal preparation, eating occasions, and expenditure coincides with major changes in residence, social networks, academic demands, and financial circumstances. Entry into higher education can intensify these transitions, creating a food environment in which established family dietary practices interact with convenience, affordability, availability, time constraints, and increasing individual responsibility for food choice [1,2]. Dietary behaviours adopted during this period may also persist beyond university, making young adults an important population for nutritional surveillance and preventive strategies [3].

University students should therefore not be viewed simply as a younger subset of the general adult population. UK and international studies have identified substantial heterogeneity in meal patterns, food choice, dietary quality, and nutrient intake among students [4,5]. This heterogeneity may be particularly important in universities with geographically diverse student populations, where previous food environments and dietary practices interact with a shared university and local food environment. At the same time, broad geographical categories cannot capture ethnicity, cultural identity, religion, socioeconomic circumstances, migration history, or acculturation and should not be interpreted as proxies for those characteristics.

Micronutrient assessment provides a particularly informative perspective on dietary quality because apparently sufficient total food or energy intake does not necessarily ensure an optimal distribution of individual vitamins. Vitamins participate in diverse physiological processes, including energy metabolism, haematopoiesis, one-carbon metabolism, antioxidant defence, immune function, vision, neurological function, and skeletal health [6]. Furthermore, the foods contributing individual vitamins vary markedly. Similar overall energy intakes may therefore coexist with very different vitamin profiles depending on the underlying foods consumed.

The interpretation of vitamin intake is nevertheless more complex than simply comparing every nutrient with a single recommended value. Dietary reference systems contain conceptually different values, including the UK lower reference nutrient intake (LRNI) and reference nutrient intake (RNI), and the European Food Safety Authority (EFSA) average requirement (AR), population reference intake (PRI), and adequate intake (AI). These quantities have different derivations and intended interpretations and are not interchangeable as individual diagnostic thresholds [7,8]. In particular, intake below an RNI, PRI, AI, or other population benchmark should not automatically be described as nutritional deficiency. Matching the measured nutrient variable precisely to the applicable reference definition is therefore essential.

Vitamin D provides a prominent example of this problem. Dietary sources are relatively restricted, and UK recommendations recognise the importance of sources beyond habitual unfortified food intake for maintaining vitamin D status [9]. Studies of UK university students and younger adults have repeatedly reported relatively low dietary vitamin D intake [10,11,12]. However, low dietary intake relative to the RNI is not equivalent to biochemical vitamin D deficiency, because vitamin D status additionally reflects cutaneous synthesis, season, skin pigmentation, outdoor exposure, and supplement use.

Folate presents a different interpretive challenge. Natural food folates and folic acid from fortified foods or supplements differ in bioavailability, and reference frameworks may consequently express intake as dietary folate equivalents (DFE). Studies in young adults have identified considerable variation in folate exposure, with a potentially important lower tail of the intake distribution despite apparently acceptable cohort-level average values [10]. Distinguishing total DFE used for dietary-reference assessment from CoFID total folate used for food source attribution is therefore important, particularly because the latter may include folic acid contributed by fortified foods.

Food source information provides a complementary dimension. Nutrient totals describe the quantity consumed but not the foods through which exposure occurs. Vitamin D, for example, may be supplied through fish, eggs, meat, dairy products, cereals, or fortified foods; vitamin B12 derives principally from animal-source and fortified foods; whereas vitamin C and a substantial proportion of folate derive from plant foods [13]. Source attribution may therefore identify dietary pathways that are more directly interpretable for nutritional intervention than nutrient totals alone. As the dietary data were collected in 2016, the present investigation provides a defined historical reference for vitamin intake distributions and their food sources within this university cohort. The integrated analytical approach may also inform future investigations using comparable dietary assessment methods, without implying that the observed intakes represent current dietary patterns among UK university students.

Preliminary findings on dietary vitamin D intake in this cohort have previously been reported in conference proceedings [14]. The present study substantially extends that initial investigation by examining 11 vitamins, applying nutrient-specific dietary reference frameworks, evaluating sex- and geographical-background-related variation using energy-adjusted models with multiplicity correction and robust sensitivity analyses, and independently reconstructing the principal food-group sources of vitamin intake. This broader approach addresses complementary nutritional questions while retaining the same historical study population. The comprehensive secondary analysis was undertaken subsequently, following consolidation and quality control of the historical dietary records and the development of the statistical expertise and reproducible analytical workflows required to examine the complete vitamin profile and its food sources.

Related investigations using the same historical cohort have subsequently examined empirically derived whole-diet patterns in relation to adiposity [15] and food-group consumption and dietary-pattern differences by sex and geographical background [16]. These investigations addressed whole-diet characteristics and their associations, whereas the present study focuses specifically on vitamin intake distributions, dietary-reference comparisons, and vitamin-specific food source attribution. A comparison of the research objectives and principal findings of these investigations is provided in Supplementary Table S1 (Panel A).

Accordingly, the objectives of this study were to: characterise the distributions of dietary vitamins A, D, E, K1, B1, B2, B3, B6, B9, B12, and C; describe vitamin intake in relation to appropriate UK and European dietary reference values; investigate sex- and geographical-background-related differences after accounting for total energy intake and multiplicity; and identify the principal food-group contributors using an independently reconstructed food source framework. These objectives were addressed through an exploratory investigation designed to identify vitamin-specific nutritional profiles, demographic differences, and their principal dietary sources.

## 2. Materials and Methods

### 2.1. Study Design, Setting, and Participants

This historical cross-sectional study examined dietary data collected from the university component of a wider nutritional and biomonitoring investigation conducted at De Montfort University (DMU), Leicester, UK, during the 2015/2016 academic year. Participant assessments contributing to the present analysis were undertaken following the institutional ethical approval granted on 11 January 2016, with data collection continuing until mid-March 2016, before the Easter vacation. The exact calendar date of the first participant assessment could not be verified from the available historical study documentation.

Students were recruited through complementary university-based approaches, including short presentations during scheduled teaching activities, announcements through Blackboard virtual-learning environments, posters and other study materials displayed on campus, and dissemination through student and course representatives [17]. Because these recruitment routes overlapped, a reliable denominator for all students exposed to study information could not be established, and a formal response rate was not calculated. The available historical records also do not permit reliable reconstruction of the numbers initially approached, screened, or excluded before establishment of the final analytical cohort.

Interested students received written information about the study and were offered flexible face-to-face appointments at the Biomedical and Environmental Health Research Laboratory, Leicester School of Allied Health Sciences, DMU. A £5 voucher was provided under an approved protocol amendment in recognition of the time required for participation. Participation was voluntary, and written informed consent was obtained before study procedures were undertaken.

Eligibility for the university component required enrolment as a DMU student, age 18–22 years, written informed consent, and fulfilment of the general-health criteria of the parent study. The present nutritional analysis additionally required dietary records of sufficient completeness and internal interpretability to support quality-controlled processing. The final analytical cohort comprised 111 participants.

The study was conducted in accordance with the Declaration of Helsinki and was approved by the Faculty Research Ethics Committee, Faculty of Health and Life Sciences, De Montfort University (Ref. 1674; 11 January 2016). Subsequent protocol amendments were approved through the institutional ethics process. Recruitment, informed consent, and all study procedures contributing data to the present analysis were undertaken following ethical approval.

### 2.2. Participant Characteristics and Geographical Background

Age and sex were recorded during the study assessment. Geographical background was derived from the recorded continent of birth and categorised as Africa, Asia, Europe, or Other.

Geographical background was used as a broad demographic descriptor and analytical variable. It was not interpreted as a proxy for genetic ancestry, ethnicity, cultural identity, religion, socioeconomic circumstances, migration history, or dietary acculturation.

The Other category comprised two participants. These individuals were retained in the overall descriptive analyses but excluded from formal geographical-background inference because the small group size did not support reliable parameter estimation. Consequently, geographical-background analyses were restricted to Africa, Asia, and Europe (*N* = 109).

Body weight, required for the participant-specific vitamin K1 dietary-reference comparison, was measured during the study appointment using a Tanita SC-330-S body-composition analyser (Tanita Corporation, Tokyo, Japan), following the manufacturer’s operating instructions [18].

### 2.3. Dietary Assessment and Data Processing

Recent dietary intake over approximately the preceding four weeks was assessed using an expanded semi-quantitative food-frequency questionnaire (FFQ) adapted from the EPIC-Norfolk FFQ, version 6 (CAMB/PQ/6/1205), developed within the European Prospective Investigation into Cancer and Nutrition (EPIC) programme at the University of Cambridge. The original EPIC-Norfolk FFQ assessed usual dietary intake over the preceding 12 months, and its methodology was evaluated using established dietary-assessment methods, including weighed food records and nutritional biomarkers [19,20].

The questionnaire administered in the present study retained the semi-quantitative food-frequency assessment approach and the frequency- and portion-size response structure, but the reference period was shortened from 12 months to approximately four weeks to assess recent dietary intake. Additional food and beverage items were incorporated to improve dietary coverage within the geographically diverse DMU student population, resulting in more than 130 food and beverage entries.

Participants reported food-consumption frequency using predefined response categories ranging from no or infrequent monthly intake to multiple occasions per day. Portion sizes were recorded using small, medium, and large categories relative to food-specific medium servings. The FFQ included nine predefined consumption-frequency categories. During the original dietary-data processing, a dietitian used Nutritics Research Edition, version 5.7 [21], following its food-specific portion-size and frequency-conversion procedures to translate the reported responses into estimated food consumption (g/week). Small, medium, and large portions were assigned food-specific serving weights rather than a single fixed gram value applied uniformly across all foods. The additional food and beverage items were processed within the same dietary-assessment workflow as the core questionnaire items. The food list covered cereals and grains, vegetables, pulses, fruit, meat and meat products, poultry, dairy products and alternatives, fish and seafood, mixed dishes, beverages, and other commonly consumed foods.

Additional food items were incorporated to broaden dietary coverage within the university setting. These additions were intended to capture dietary heterogeneity rather than to assign particular foods or dietary behaviours to participants on the basis of their geographical background. Food-composition interpretation was informed by established UK resources, including McCance and Widdowson’s *The Composition of Foods* [22]. The FFQ also included supplementary questions addressing vitamin, mineral, and multivitamin/multimineral supplement use, including reported frequency and dose. These questions were distinct from the food-frequency entries used for the Nutritics-based nutrient assessment.

Completed questionnaires were reviewed for sufficient completeness and internal interpretability. Dietary records were initially entered and exported using Nutritics Research Edition, version 5.7 (Nutritics Ltd., Dublin, Ireland), between May and June 2018. These records provided the original participant-level nutrient estimates and food-level consumption data. The archived outputs were subsequently consolidated and quality-controlled before implementation of the definitive statistical analyses, while retaining the original Nutritics nutrient estimates as the primary dietary exposure variables.

In the retained source database, estimated food consumption was expressed in grams per week. Daily food intake was therefore calculated by dividing weekly consumption by seven. This conversion was applied once only. Raw and derived variables were maintained separately during quality control.

The quality-controlled participant-level Nutritics vitamin totals were retained as the definitive dietary exposure variables for descriptive analyses, dietary-reference comparisons, and demographic modelling. The original food-level consumption records were used separately for the reconstruction of vitamin-specific food sources, as described in Section 2.6. No separately quantified vitamin or mineral supplement contributions from the supplementary FFQ questions were added to the participant-level nutrient estimates. Accordingly, the analysed vitamin intakes reflect the recorded foods, beverages, and nutritional preparations processed in Nutritics, rather than total dietary intake inclusive of independently reported vitamin or mineral supplements. Folate DFE estimates may include folic acid contributed by fortified food products.

### 2.4. Vitamin Variables

Eleven vitamin variables were evaluated: vitamin A, expressed as total retinol equivalents (RE); vitamin D; vitamin E; vitamin K1 (phylloquinone); thiamin (vitamin B1); riboflavin (vitamin B2); niacin, expressed as total niacin equivalents (NE); vitamin B6; folate, expressed as total dietary folate equivalents (DFE); vitamin B12; and vitamin C. Vitamin A was obtained from the original Nutritics total retinol-equivalent (RE) field, which incorporates preformed retinol and the vitamin A activity of provitamin A carotenoids. Niacin was obtained from the total niacin-equivalent (NE) field, incorporating preformed niacin and the contribution of tryptophan, conventionally expressed as 1 mg NE per 60 mg of tryptophan. For folate, the original Nutritics dietary folate-equivalent (DFE) variable was retained, accounting for natural food folate and the higher bioavailability of folic acid from fortified foods. These software-derived nutrient totals were preserved as the participant-level exposure variables rather than recalculated from individual nutrient components.

The Nutritics total vitamin E variable was retained for descriptive and inferential analysis. The historical dataset additionally contained a separate α-tocopherol field. This variable was evaluated during quality control but was not assumed to represent complete dietary α-tocopherol exposure for reference-value classification. Accordingly, total vitamin E and α-tocopherol were not treated as interchangeable analytical variables.

For dietary-reference analyses, folate intake was expressed as total dietary folate equivalents (DFE) obtained from Nutritics. For food source attribution, the corresponding CoFID variable was FOLT (total folate, µg/100 g), which may include synthetic folic acid where represented in fortified food products. CoFID total folate is therefore not synonymous with natural folate and is not directly equivalent to DFE. These nutrient definitions were maintained separately in the analysis and interpretation.

### 2.5. Dietary Reference Framework

Dietary reference values were selected according to the nutritional definition of each vitamin variable and the intended purpose of the comparison. UK lower reference nutrient intakes (LRNIs) were used for seven vitamins to characterise the lower end of the intake distribution, whereas the UK reference nutrient intake (RNI) was selected for vitamin D as the principal UK dietary benchmark. For folate, EFSA average requirement (AR) and population reference intake (PRI) values were selected because they are expressed in dietary folate equivalents (DFE), corresponding to the Nutritics total-DFE variable, rather than using the UK folate RNI of 200 µg/day, which is not expressed on the same DFE basis. Other reference values were applied according to their nutrient-specific definitions and interpretive purposes, as detailed below.

For vitamin A, thiamin, riboflavin, niacin, vitamin B6, vitamin B12, and vitamin C, UK LRNI values were used to identify the extreme lower end of the intake distribution [7]. The number of vitamins below the UK LRNI per participant was calculated using these seven vitamins only.

Sex-specific LRNI thresholds were applied to vitamin A. Participant-specific thresholds were calculated for thiamin and niacin according to individual energy intake and for vitamin B6 according to individual protein intake.

Vitamin D was compared with the UK RNI of 10 µg/day [9].

Total folate DFE was compared with the EFSA AR of 250 µg DFE/day and the PRI of 330 µg DFE/day [23].

Vitamin K1 was compared descriptively with the UK safe-intake benchmark of approximately 1 µg/kg body weight/day and the EFSA AI of 70 µg/day [7,24].

The EFSA AI for vitamin E is expressed specifically in relation to α-tocopherol [25]. Because the available historical α-tocopherol field could not be shown with sufficient confidence to represent complete dietary α-tocopherol exposure comparable with this AI, no participant-level classification was performed. Vitamin E was therefore designated NOT CLASSIFIED for this comparison.

LRNI, RNI, AR, PRI, AI, and safe-intake values have different interpretive meanings. Proportions below an AI or safe-intake benchmark were treated as descriptive comparisons and were not interpreted as estimates of individual inadequacy or biochemical deficiency. Similarly, dietary intake below an RNI or PRI was not treated as a clinical diagnosis.

For proportions below dietary reference values, 95% confidence intervals were calculated using the Wilson score method [26].

### 2.6. Food Source Reconstruction and Calibration

An independent food source reconstruction was undertaken to characterise the foods underlying the participant-level vitamin exposures. This reconstruction was used for source attribution and did not replace the original Nutritics vitamin totals used in the primary participant-level analyses.

The retained food-level dietary dataset comprised 441 distinct FoodName entries. Daily food-consumption estimates were linked through a reviewed crosswalk to corresponding nutrient-composition entries from the UK Composition of Foods Integrated Dataset (CoFID) [22]. Each food was assigned to an analytical food group for vitamin source attribution. These groups were designed to preserve nutritionally interpretable food source categories and were not assumed to be identical to the grouping structure used in previous dietary-pattern analyses of the same cohort. The independent food source reconstruction was undertaken subsequently using CoFID 2021 composition data. The initial food-composition matching and food source group assignments were performed by the corresponding author (A.P.-F.) and subsequently reviewed by a clinical research dietitian who had not participated in the initial assignments. Historical food descriptions, including branded products where available, were matched to appropriate composition entries using the documented food characteristics and available nutritional information, without assuming identical commercial formulations across different years. This procedure provided a separate framework for identifying vitamin-specific food sources while preserving the original Nutritics-derived nutrient estimates used in the participant-level statistical analyses.

For vitamin E, the CoFID ‘VITE’ field and the Nutritics total vitamin E field were both defined in terms of α-tocopherol equivalents, supporting their use within the comparative food source reconstruction. The individual α-tocopherol fraction available in the original Nutritics export was retained as a separate variable and was not considered interchangeable with total vitamin E activity.

For participant *i*, food *f*, and vitamin *v*, raw reconstructed vitamin contribution was calculated from daily food intake and the corresponding CoFID vitamin concentration. Raw food-level contributions were then summed to obtain participant-level reconstructed totals.

Because Nutritics and CoFID represent distinct food-composition systems and may differ in food matching, formulation, fortification, and nutrient values, the independent CoFID reconstruction was compared with the original participant-level Nutritics totals before calibration. Agreement was quantified using Pearson and Spearman correlations, median absolute relative differences, and the proportion of cohort food grams with usable composition data.

For source attribution, participant-specific calibration factors were subsequently calculated as the ratio of the original Nutritics vitamin total to the corresponding reconstructed CoFID total. Food-level CoFID contributions were multiplied by this factor before aggregation into analytical food groups. Thus, calibration preserved the relative food source distribution generated by the independent reconstruction while aligning participant-level totals with the definitive Nutritics exposure.

Food-group percentage contributions were then calculated from calibrated cohort-level nutrient totals. The stability of food source structure before and after calibration was evaluated using Pearson correlation, Spearman correlation, and overlap of the five highest-ranked food groups.

For folate, food source contributions were reconstructed using the CoFID FOLT field, representing total food folate. Fortified foods recorded in the original dietary dataset, including breakfast cereals, were retained in their corresponding food groups. As the available composition data did not consistently distinguish naturally occurring folate from added folic acid, these contributions were not separated. The FOLT-derived food source estimates were calibrated against the original Nutritics natural-folate totals rather than total DFE. This calibration provided a scaling procedure for food source attribution but did not establish equivalence between CoFID total folate and Nutritics natural folate. Consequently, the reconstructed percentages describe CoFID total-folate-based food source patterns rather than natural-folate-only contributions.

Vitamin K1 was treated separately because usable CoFID composition data covered only 47.7% of cohort food grams. Two invalid negative K1 composition values arising from derived oil-substitution entries were treated as unavailable (NA), rather than zero, before K1-specific recalibration. The original Nutritics dataset contained participant-level K1 estimates for all 111 individuals; however, the archived files did not provide sufficient food-level nutrient-composition information to quantify the underlying completeness of Nutritics K1 coverage independently. Given this uncertainty and the median absolute relative difference of 23.2% between raw CoFID reconstruction and Nutritics totals, K1 food source attribution was designated exploratory and excluded from the principal food source table and heatmap.

### 2.7. Statistical Analysis

All definitive statistical analyses were conducted using R version 4.4.2 (R Foundation for Statistical Computing, Vienna, Austria) [27].

Continuous vitamin variables were summarised using mean ± standard deviation (SD), median [interquartile range (IQR)], range, and 5–95th percentile interval. Categorical variables were reported as *n* (%). Reported total energy and protein intakes were examined for completeness, distribution, and potential extreme observations. An exploratory upper Tukey fence (Q3 + 1.5 × IQR) was used to identify unusually high energy-intake records for further assessment. Energy-intake-to-basal-metabolic-rate ratios were also examined descriptively. Individual misreporting classifications were not assigned because sufficiently detailed participant-level physical-activity information was unavailable.

For adjusted demographic comparisons, vitamin intakes were expressed in their original daily units (µg/day for vitamins A, D, K1, B9, and B12; mg/day for vitamins E, B1, B2, B3, B6, and C). Each outcome was transformed using log1p [ln(1 + intake)] to reduce positive skewness while accommodating potential zero intakes, and subsequently standardised. The standardisation mean and standard deviation were calculated separately for each vitamin using the complete cohort (*N* = 111), before restricting geographical-background analyses to 109 participants. Thus, sex and geographical-background models used the same standardised outcome scale for each vitamin, and regression coefficients represent adjusted differences in standard deviation units.

The primary sex model for each vitamin included sex and log-transformed total energy intake. Sex analyses included the complete cohort (*N* = 111).

Primary geographical-background models included geographical background and log-transformed total energy intake. Formal analyses compared Africa, Asia, and Europe and therefore included *N* = 109. Global tests were evaluated before pairwise contrasts.

Multiple testing was controlled using the Benjamini–Hochberg false-discovery-rate (BH-FDR) procedure within the corresponding prespecified inferential families [28]. Interpretation prioritised adjusted effect estimates, 95% confidence intervals, and FDR-adjusted *q*-values rather than isolated nominal *p*-values.

Fully adjusted sensitivity models additionally incorporated age and the complementary demographic variable: sex. Sensitivity models included age and geographical background, whereas geographical-background sensitivity models included age and sex. HC3 heteroscedasticity-consistent covariance estimation was used for these sensitivity analyses [29].

An additional exploratory sensitivity analysis assessed the influence of unusually high reported energy intakes by excluding the five participants (IDs 24, 26, 36, 55, and 67) exceeding the upper Tukey fence of 5272.64 kcal/day. The original energy-adjusted sex and geographical-background models were repeated using the reduced samples (*N* = 106 and *N* = 104, respectively). Outcome standardisation was recalculated within the reduced cohort before geographical restriction, following the original analytical procedure. The same BH-FDR correction families were retained (11 sex comparisons, 11 global geographical tests, and 33 pairwise geographical contrasts). All five participants remained included in the primary analyses.

The analytical sample was fixed by the number of participants from the parent study with complete quality-controlled vitamin data; no prospective sample-size calculation was undertaken specifically for the present secondary analysis.

The study is reported in accordance with the principles of the STROBE statement for observational cross-sectional studies [30].

## 3. Results

### 3.1. Study Population and Distribution of Vitamin Intakes

The analytical cohort comprised 111 students aged 18–22 years. Mean participant age was 20.43 ± 1.14 years. Seventy-eight participants were female (70.3%), and 33 were male (29.7%). Geographical background was classified as Africa for 41 participants (36.9%), Asia for 41 (36.9%), Europe for 27 (24.3%), and Other for 2 (1.8%). Mean reported energy intake was 2524.10 ± 1256.09 kcal/day, and mean protein intake was 118.29 ± 62.31 g/day, with complete data for all 111 participants. Energy intake ranged from 890.31 to 8862.48 kcal/day, with five observations exceeding the exploratory upper Tukey fence of 5272.64 kcal/day. These observations were retained in the primary analytical dataset. Detailed demographic, anthropometric, and dietary characteristics by geographical background, including sex distribution and missing-data information, are presented in Supplementary Table S1 (Panel B).

Daily vitamin intake distributions are presented in Table 1. Sex- and geographical-background-specific descriptive distributions are provided in Supplementary Tables S2 and S3, respectively.

Mean vitamin A intake was 1013.74 ± 739.81 µg RE/day, with a median of 875.47 µg/day [IQR 561.72–1210.53]. Mean vitamin D intake was 3.96 ± 3.13 µg/day and median intake was 3.06 µg/day [1.78–5.21]. Mean vitamin E intake was 11.58 ± 6.49 mg/day, while mean vitamin K1 intake was 102.18 ± 95.29 µg/day.

Mean thiamin and riboflavin intakes were 2.15 ± 1.08 and 2.43 ± 1.43 mg/day, respectively. Mean niacin intake was 58.96 ± 30.65 mg NE/day and mean vitamin B6 intake was 3.10 ± 1.73 mg/day.

Mean folate intake was 387.74 ± 208.50 µg DFE/day, with a median of 341.71 µg/day [227.65–499.46]. Mean vitamin B12 intake was 9.57 ± 5.90 µg/day. Vitamin C had a mean intake of 181.86 ± 132.25 mg/day and a median of 154.93 mg/day [91.69–227.28].

### 3.2. Vitamin Intakes Relative to Dietary Reference Values

**Figure 1:**
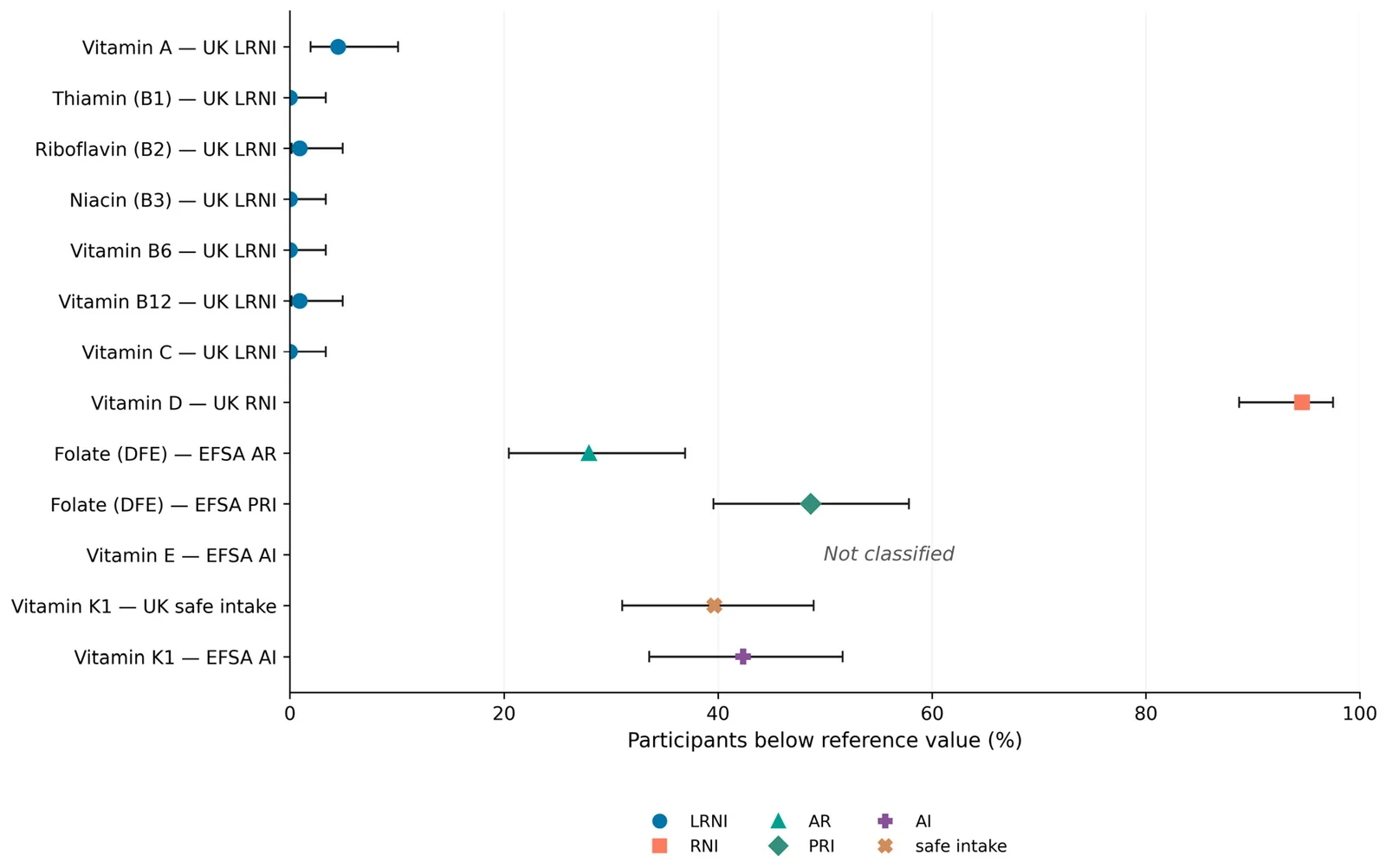
Percentage of participants with vitamin intakes below selected dietary reference values, with 95% confidence intervals. The reference systems are identified for each vitamin: UK LRNI (vitamins A, B1, B2, B3, B6, B12, and C), UK RNI (vitamin D), EFSA AR and PRI (folate), and UK safe intake and EFSA AI (vitamin K1). Vitamin E was not classified against the EFSA AI. Because these reference values have different nutritional definitions and interpretative purposes, percentages below the respective thresholds are not directly comparable as measures of nutritional inadequacy. The corresponding reference values, classifications, and participant counts are summarised in Table 2.

Selected dietary-reference comparisons are presented in Table 2 and visualised in Figure 1.

Intakes below UK LRNI thresholds were uncommon. Five participants had vitamin A intake below the sex-specific UK LRNI (4.5%; 95% CI 1.9–10.1%). One participant was below the LRNI for riboflavin (0.9%; 0.2–4.9%), and one for vitamin B12 (0.9%; 0.2–4.9%). No participant was below the corresponding LRNI for thiamin, niacin, vitamin B6, or vitamin C.

When the seven prespecified LRNI vitamins were considered jointly, 104 participants (93.7%) had no vitamin below an LRNI and 7 (6.3%) had one vitamin below an LRNI. No participant had two or more of these vitamins below the relevant LRNI. This distribution is shown in Supplementary Figure S1.

A markedly different profile was observed for vitamin D. Overall, 105 of 111 participants had dietary vitamin D intake below the UK RNI of 10 µg/day, corresponding to 94.6% (95% CI 88.7–97.5%).

For folate, 31 participants (27.9%; 95% CI 20.4–36.9%) had total DFE intake below the EFSA AR of 250 µg/day and 54 (48.6%; 39.6–57.8%) were below the PRI of 330 µg/day.

Vitamin E was not classified against the EFSA AI because the comparability and completeness of the historical α-tocopherol variable could not be established.

For vitamin K1, 44 participants (39.6%; 31.0–48.9%) had intake below the participant-specific UK safe-intake benchmark and 47 (42.3%; 33.6–51.6%) were below the EFSA AI. These vitamin K1 comparisons are descriptive and exploratory because the completeness of the underlying historical food-composition data could not be independently established; they should not be interpreted as estimates of individual nutritional inadequacy.

### 3.3. Adjusted Differences by Sex and Geographical Background

Primary adjusted sex and geographical-background comparisons are presented in Table 3. Complete primary analyses are provided in Supplementary Tables S4–S6, and fully adjusted HC3 sensitivity analyses in Supplementary Tables S7–S9. Complete primary and HC3 forest plots are shown in Supplementary Figures S2 and S3, respectively.

Following adjustment for total energy intake and BH-FDR correction, vitamin A was the only vitamin showing an FDR-significant sex difference. Males had lower adjusted vitamin A intake than females (adjusted difference −0.484 SD; 95% CI −0.796 to −0.172; *p* = 0.003; *q* = 0.029).

Nominal sex differences were observed for vitamin E, vitamin K1, and vitamin B6, but these did not remain statistically significant after correction for multiple testing. No FDR-significant sex difference was observed for vitamin D, thiamin, riboflavin, niacin, folate, vitamin B12, or vitamin C.

For geographical background, global tests remained FDR-significant for vitamin E (*q* = 0.034), vitamin K1 (*q* = 0.034), niacin (*q* = 0.034), vitamin B6 (*q* = 0.034), and vitamin B12 (*q* = 0.049).

Pairwise contrasts indicated higher adjusted vitamin E intake in participants from Asia than from Africa (+0.359 SD; 95% CI 0.102–0.616; *q* = 0.044). Vitamin K1 intake was higher in the European than in the African group (+0.583 SD; 0.182–0.985; *q* = 0.044). The vitamin K1 contrast should nevertheless be interpreted as exploratory because uncertainty remains regarding the completeness of the original nutrient-composition data.

Niacin intake was lower in participants from Asia than from Africa (−0.319 SD; −0.558 to −0.081; *q* = 0.044) and lower in Asia than Europe (−0.372 SD; −0.638 to −0.106; *q* = 0.044).

Vitamin B6 showed a similar pattern, with lower adjusted intake in Asia than in Africa (−0.355 SD; −0.619 to −0.090; *q* = 0.044) and in Asia than in Europe (−0.453 SD; −0.748 to −0.157; *q* = 0.044).

Vitamin B12 intake was lower in Asia than in Africa (−0.413 SD; −0.722 to −0.104; *q* = 0.044). The corresponding Asia–Europe contrast did not remain FDR-significant.

The fully adjusted HC3 sensitivity analyses broadly supported the global geographical-background results for vitamins E and K1, niacin, and vitamin B6. In contrast, the global vitamin B12 result was attenuated from *q* = 0.049 in the primary model to *q* = 0.072 in the HC3 sensitivity analysis and should therefore be interpreted as less robust. In the additional sensitivity analysis excluding five participants with unusually high reported energy intakes, the adjusted sex difference for vitamin A remained FDR-significant (*q* = 0.0187). Global geographical-background differences also remained significant for vitamins E, K1, and B6 (*q* = 0.0420 for each). However, the global niacin association no longer met the BH-FDR threshold (*q* = 0.0507), and the vitamin B12 association was further attenuated (*q* = 0.1080). None of the seven geographical pairwise contrasts that were FDR-significant in the primary analyses retained significance after exclusion of these observations. These findings indicate greater sensitivity of the specific geographical contrasts to high-energy-intake records than of the vitamin A sex association or the global geographical associations for vitamins E, K1, and B6.

For completeness, the full dietary-reference comparison set, including additional UK RNI comparisons not selected for the principal reference-value figure, is provided in Supplementary Table S10.

### 3.4. Major Food-Group Contributors and Reconstruction Validation

**Figure 2:**
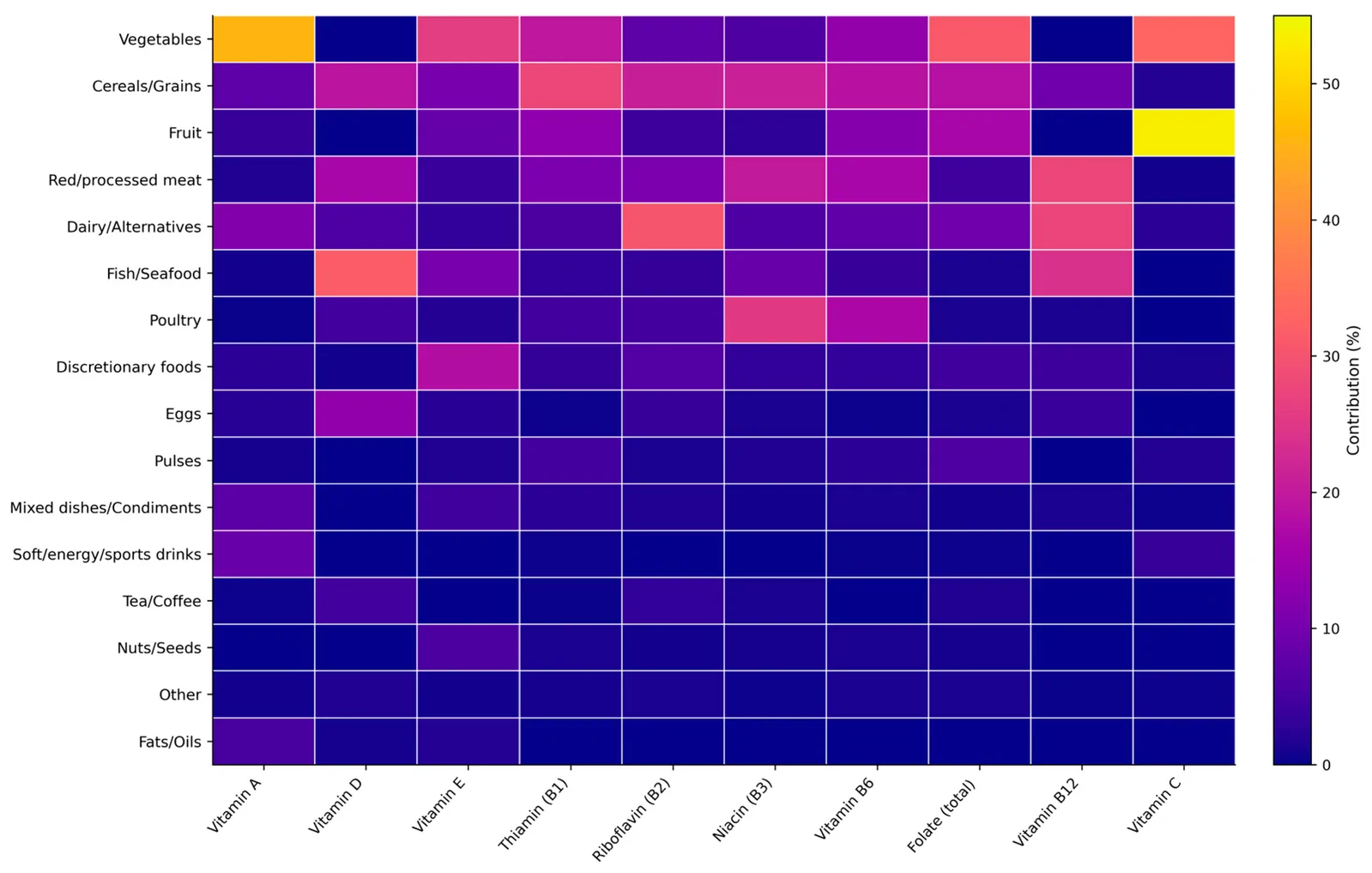
Major food-group sources of vitamin intake based on calibrated percentage contributions from the CoFID-based reconstruction. Folate represents CoFID total folate (FOLT), which may include folic acid from fortified foods. Vitamin K1 is excluded because food source attribution was treated as exploratory owing to incomplete composition coverage.

The overall calibrated food source landscape is shown in Figure 2, and the five highest-ranked contributors for each vitamin are summarised in Table 4.

Vitamin A intake was dominated by vegetables, which contributed 45.9% of the calibrated total. Dairy/alternatives contributed 11.6%, soft/energy/sports drinks 8.8%, cereals/grains 7.6%, and mixed dishes/condiments 7.1%. Together, the five highest-ranked groups accounted for 81.0% of reconstructed vitamin A intake.

Fish/seafood was the largest vitamin D source, contributing 31.8%, followed by cereals/grains (19.1%), red/processed meat (16.7%), eggs (13.5%), and dairy/alternatives (6.0%). These five groups accounted for 87.2% of reconstructed vitamin D intake.

Vegetables provided the largest contribution to vitamin E intake (26.1%), followed by discretionary foods (18.3%), cereals/grains (10.8%), fish/seafood (10.5%), and fruit (8.5%).

Cereals/grains were the principal thiamin source (28.1%), while dairy/alternatives were the largest contributor to riboflavin (30.3%). Poultry was the largest niacin contributor (25.5%), followed by cereals/grains (21.1%) and red/processed meat (20.1%).

Vitamin B6 was supplied by a relatively distributed set of food groups, led by cereals/grains (18.5%), poultry (16.9%), red/processed meat (16.7%), vegetables (13.9%), and fruit (12.2%).

For CoFID total folate, vegetables accounted for 31.0%, cereals/grains for 18.3%, fruit for 16.7%, dairy/alternatives for 9.4%, and pulses for 6.1%.

Vitamin B12 was supplied predominantly by red/processed meat (27.8%), dairy/alternatives (27.5%), and fish/seafood (23.9%). Together with cereals/grains and discretionary foods, the five highest-ranked groups accounted for 93.0% of reconstructed vitamin B12 intake.

Vitamin C showed the most concentrated plant-food source profile. Fruit contributed 53.4% and vegetables 32.9%; these two groups alone therefore supplied 86.3% of reconstructed vitamin C intake.

Agreement between the independent raw CoFID reconstruction and the corresponding original Nutritics participant-level totals is reported in Supplementary Table S11 and visualised in Supplementary Figure S4. Pearson correlations ranged from 0.935 to 0.993 for most vitamins. Vitamin B6 showed lower but still positive agreement (r = 0.826). Composition coverage exceeded 88% for all principal food source vitamins and was generally above 95%, with vitamin K1 representing the clear exception at 47.7%.

The relative food-group source structure was highly stable before and after calibration (Supplementary Table S12). Pearson correlations between raw and calibrated food-group percentage profiles ranged from 0.997 to 1.000, and the same five highest-ranked food groups were retained for every vitamin.

The exploratory vitamin K1 attribution is presented separately in Supplementary Table S13. Vegetables dominated this reconstruction, but the limited composition coverage prevents its treatment as equivalent in reliability to the principal food source analyses.

### 3.5. Integrated Summary of Adjusted Demographic Differences

**Figure 3:**
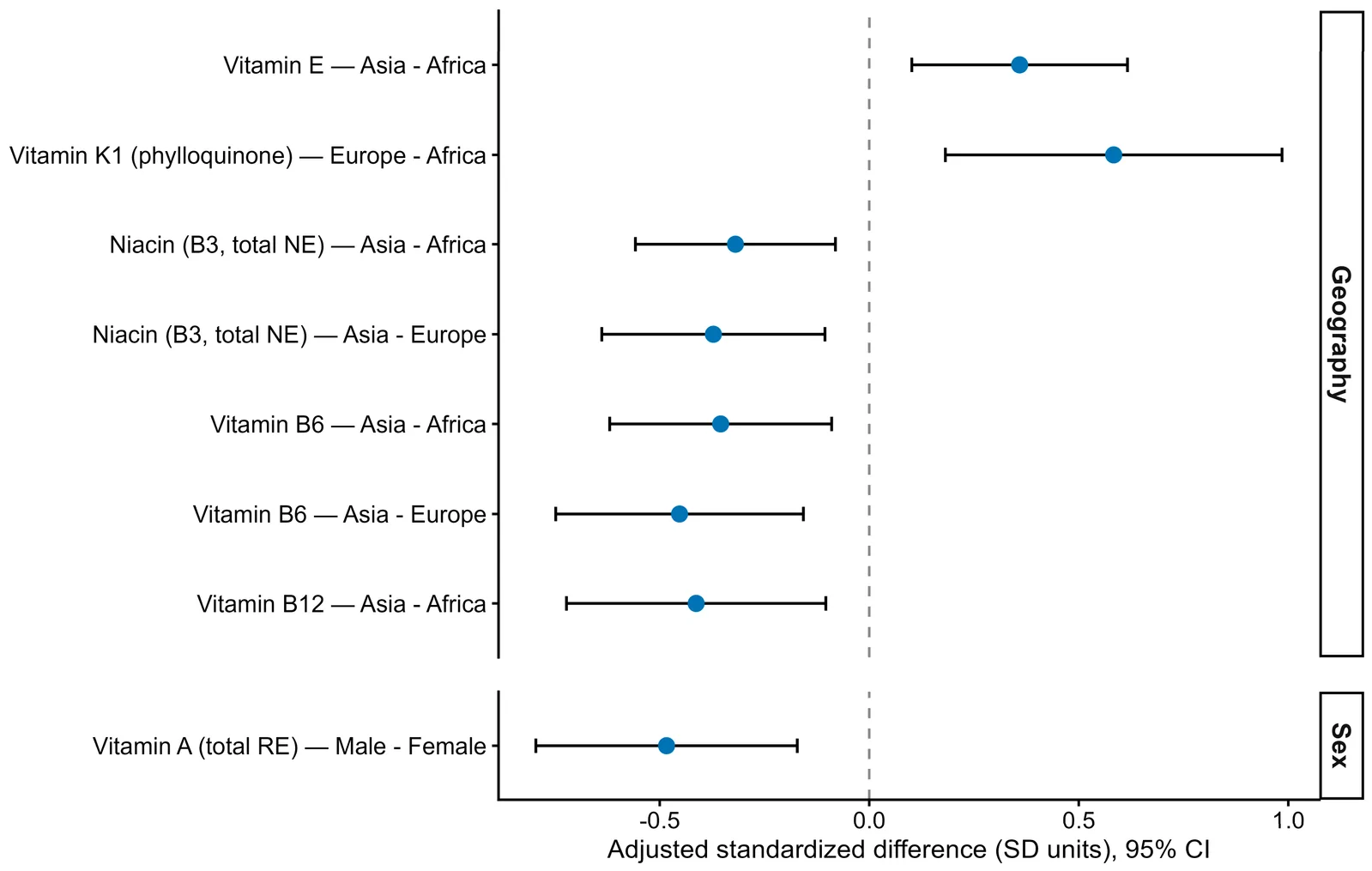
Primary adjusted standardized differences that remained significant after Benjamini–Hochberg false-discovery-rate correction. Sex estimates are energy-adjusted male–female differences. Geographical-background estimates are pairwise contrasts from the primary energy-adjusted models. Points show adjusted standardized differences, and horizontal bars show 95% confidence intervals; complete numerical results are reported in Table 3 and Supplementary Table S6. Blue dots represent adjusted standardized differences, horizontal bars indicate 95% confidence intervals, and the dashed vertical line indicates zero difference. Vitamin K1 results are shown for transparency but should be interpreted as exploratory because the completeness of the historical food-composition data could not be independently verified. The seven FDR-significant geographical pairwise contrasts shown in the primary analysis did not retain BH-FDR significance in the additional sensitivity analysis excluding five high-energy-intake observations.

The adjusted contrasts that remained significant after BH-FDR correction are summarised visually in Figure 3.

The figure highlights the comparatively restricted sex-related signal, confined to lower adjusted vitamin A intake among males, alongside the broader geographical-background pattern involving vitamins E and K1, niacin, vitamin B6, and vitamin B12. As noted above, vitamin B12 was less robust in the fully adjusted HC3 sensitivity analysis.

## 4. Discussion

### 4.1. Principal Findings

This study identified a strongly nutrient-specific vitamin profile among young adults attending an English university. The central finding was not widespread extremely low intake across the vitamin panel. For the seven vitamins evaluated against UK LRNIs, 93.7% of participants were below none of the prespecified LRNIs, and no participant was below two or more. Instead, the most prominent departures from the dietary-reference framework concerned vitamin D and folate.

Dietary vitamin D intake was below the UK RNI in 94.6% of participants. Folate showed a different but still important pattern, with 27.9% of participants below the EFSA AR and 48.6% below the PRI. These findings coexisted with comparatively low frequencies below the UK LRNIs for vitamins A, B1, B2, B3, B6, B12, and C.

A second important finding was that the food source patterns underlying these vitamin totals differed substantially between nutrients. Fish/seafood was the leading reconstructed source of vitamin D; red/processed meat, dairy/alternatives, and fish/seafood dominated vitamin B12; vegetables, cereals/grains, and fruit were the principal reconstructed total-folate sources; and fruit and vegetables supplied the great majority of vitamin C.

Finally, demographic heterogeneity was more apparent across geographical-background groups than between sexes. Vitamin A was the only sex comparison that survived multiplicity correction, whereas vitamins E and K1, niacin, vitamin B6, and vitamin B12 showed FDR-significant global geographical-background variation in the primary analyses. The vitamin B12 finding was less stable under fully adjusted HC3 sensitivity analysis.

Taken together, these results illustrate why a vitamin profile cannot be reduced to either a single concept of “micronutrient adequacy” or a set of cohort means. Interpretation requires consideration of the complete intake distribution, the specific reference framework applicable to each vitamin, and the foods supplying that exposure.

### 4.2. Vitamin D: A Widespread Dietary Reference-Value Shortfall

Vitamin D showed the clearest departure from the selected dietary-reference framework. Mean dietary intake was 3.96 µg/day and median intake was 3.06 µg/day, while 105 of 111 participants (94.6%) had intake below the UK reference nutrient intake (RNI) of 10 µg/day. This pronounced shortfall is consistent with previous investigations in UK university and young-adult populations. Farhat et al. (2019), in a cross-sectional study of 542 university students aged 18–25 years in northwest England, reported mean dietary vitamin D intakes of 4.44 µg/day in males and 5.04 µg/day in females [10]. Similarly, Dong et al. (2022) reported a mean intake of 4.6 ± 3.9 µg/day among 41 university students aged 18–29 years in Coventry, of whom only 12.1% reached the recommended 10 µg/day [11]. These findings are consistent with national dietary surveillance and subsequent UK Scientific Advisory Committee on Nutrition (SACN) assessments identifying vitamin D as a nutrient for which dietary intake frequently remains below the recommended level [9,31,32].

The similarity between these findings is noteworthy because the present cohort was geographically diverse and assessed using an adapted food-frequency questionnaire, whereas other investigations employed different dietary-assessment methods and sampling approaches. Although direct numerical comparisons of prevalence are not warranted, the recurring observation of relatively low dietary vitamin D intake across different UK young adult populations suggests that this finding is not unique to the present cohort.

The interpretation of these results must nevertheless distinguish dietary intake from nutritional status. Intake below the UK RNI does not establish biochemical vitamin D deficiency. Circulating 25-hydroxyvitamin D [25(OH)D] concentrations are influenced not only by dietary intake but also by cutaneous synthesis, season, skin pigmentation, outdoor exposure, clothing, supplement use and other individual factors [9,11]. This distinction is particularly relevant because the present cohort was assessed between January and March, a period during which cutaneous vitamin D synthesis is generally limited at UK latitudes. However, vitamin D biomarkers were not available for the present analysis, and the findings therefore characterise dietary exposure rather than biochemical vitamin D status.

The food source analysis provides additional practical context for understanding these patterns. Fish and seafood represented the principal reconstructed dietary source of vitamin D (31.8%), followed by cereals and grains (19.1%), red and processed meat (16.7%), eggs (13.5%) and dairy products and alternatives (6.0%). Comparable findings have been reported in European population studies. In the Hellenic National Nutrition and Health Survey, Mitsopoulou et al. [13] identified fish and shellfish as the leading contributors to vitamin D intake, accounting for approximately 29.8%, followed by other animal-derived and commonly consumed food groups. The close similarity in the contribution from fish and seafood is particularly interesting given the differences in geographical setting, habitual dietary patterns and dietary-assessment methodology. The broader distribution of contributions across cereals, meat, eggs and dairy in the present cohort illustrates why strategies aimed at improving dietary vitamin D intake cannot focus exclusively on fish consumption.

The contribution of cereals and other widely consumed foods also highlights the potential importance of food composition and fortification. However, their contribution in the present reconstruction should not automatically be attributed to fortification, because the historical food records do not establish the fortification status of every consumed product. Analyses of UK dietary-survey data have distinguished the contributions of the base diet, voluntarily fortified foods and dietary supplements to micronutrient intakes [33]. Allen et al. [34], using UK dietary-survey data and modelling different fortification scenarios, demonstrated that vitamin D fortification of staple foods, particularly wheat flour, could substantially improve estimated population vitamin D intakes and status among groups at risk of low exposure. More recent UK assessments have likewise identified food fortification as a potentially useful population-level strategy for improving vitamin D exposure [31]. Taken together, these findings support the importance of considering both total vitamin D intake and the foods through which it is obtained. They also suggest that improving dietary vitamin D exposure among young adults may require approaches combining appropriate food choices with broader nutritional strategies, while recognising that dietary intake alone does not determine vitamin D status.

### 4.3. Folate: Why the Distribution Matters More than the Mean Alone

Folate presented a distinct nutritional profile. Mean total folate intake was 387.74 µg DFE/day and median intake was 341.71 µg DFE/day—values that could appear relatively reassuring if considered in isolation. However, 31 participants (27.9%) had intakes below the EFSA average requirement (AR) of 250 µg DFE/day, while 54 (48.6%) were below the population reference intake (PRI) of 330 µg DFE/day. These findings illustrate the importance of examining the complete intake distribution rather than relying exclusively on cohort-level means. A mean intake above a reference value can coexist with a substantial proportion of participants below that benchmark, and the proportion below the PRI should not be interpreted as a direct estimate of nutritional inadequacy.

Previous studies have also identified considerable variation in folate intake among young adults. Farhat et al. (2019), investigating university students aged 18–25 years in northwest England, reported relatively low dietary folate intakes, particularly among female participants [10]. Derbyshire [35], using UK National Diet and Nutrition Survey data, identified variation in micronutrient intake across adult age groups. Broader European investigations have likewise demonstrated heterogeneity in micronutrient exposure between countries and population subgroups. Mensink et al. [36], analysing national dietary surveys from eight European countries, documented differences in the distribution of several micronutrients, including folate, while Rippin et al. [37] highlighted variation in nutrient intakes and dietary-assessment approaches across European populations. These findings provide a useful context for the present results, although differences in assessment methodology, food-composition data and reference frameworks limit direct comparisons of absolute intake and prevalence.

An additional consideration concerns the chemical form and bioavailability of dietary folate. Naturally occurring food folates and synthetic folic acid from fortified foods or supplements differ in bioavailability, and their contributions may therefore be expressed using dietary folate equivalents. McNulty and Scott [38] emphasised the importance of distinguishing folate intake from folate status and discussed the effects of food processing, stability and bioavailability on folate exposure. These considerations are particularly relevant when comparing studies that report natural folate, total folate or dietary folate equivalents, because these variables are not necessarily interchangeable.

In the present study, total DFE was retained for dietary-reference comparisons to match the EFSA framework. In contrast, food source attribution was based on the CoFID total-folate field (FOLT), with reconstructed contributions calibrated against the corresponding Nutritics natural-folate totals. As CoFID FOLT may include folic acid from fortified foods, these variables are not chemically identical, and calibration should not be interpreted as establishing nutritional equivalence. The resulting percentages therefore characterise the reconstructed food source distribution of CoFID total folate rather than the separate contributions of naturally occurring folate and added folic acid.

The reconstructed food source profile was dominated by vegetables (31.0%), cereals and grains (18.3%) and fruit (16.7%), which together contributed approximately two-thirds of the reconstructed CoFID total-folate source profile. Dairy products and alternatives (9.4%) and pulses (6.1%) provided additional contributions. This distribution is broadly consistent with national and European food source investigations identifying vegetables, cereals and other plant-derived foods as important sources of dietary folate [13]. The present findings therefore demonstrate how food-group composition can complement total nutrient estimates by identifying the dietary pathways underlying variation in folate exposure. In particular, the coexistence of an apparently favourable cohort mean with substantial proportions below selected EFSA reference values highlights the need to consider both the distribution of intake and the foods contributing to it.

### 4.4. Intakes Below UK LRNIs Were Uncommon, but This Does Not Demonstrate Universal Adequacy

A particularly important finding was the low prevalence of intakes below UK lower reference nutrient intake (LRNI) thresholds across the seven prespecified vitamins. No participant was below the LRNI for thiamin, niacin, vitamin B6 or vitamin C, while only one participant (0.9%) was below the corresponding LRNI for riboflavin and one (0.9%) for vitamin B12. Vitamin A showed the highest prevalence below LRNI, affecting five participants (4.5%). Overall, 104 of 111 participants (93.7%) had no vitamin below the seven selected LRNIs, while seven participants (6.3%) had one vitamin below an LRNI. No participant had two or more vitamins below these thresholds.

These findings must be interpreted within the intended meaning of the UK dietary-reference framework. The LRNI represents an intake sufficient for only a small proportion of individuals with the lowest requirements and is not intended to function as a general dietary target. Consequently, the observation that 93.7% of participants were below none of the seven examined LRNIs does not establish that the same proportion had optimal or fully adequate vitamin intake. The LRNI identifies the extreme lower end of the reference framework, whereas the reference nutrient intake (RNI) represents a substantially higher intake level intended to meet the requirements of nearly all individuals within the relevant population group [7].

The importance of distinguishing these reference categories becomes apparent when additional UK RNI comparisons are considered. For vitamin A, 34.2% of participants were below the RNI, despite only 4.5% being below the corresponding LRNI. Riboflavin showed a similar, although smaller, separation between the proportions below the two reference values. These differences are not contradictory; they arise because the thresholds have different nutritional meanings. Accordingly, the prevalence of intake below a reference value depends not only on the observed dietary distribution but also on the criterion selected for comparison.

This issue has also been highlighted in European nutritional research. Tabacchi et al. [39], in a systematic review of micronutrient adequacy assessment across Europe, identified substantial variation in the approaches used to evaluate dietary adequacy. Mensink et al. [36] similarly demonstrated the importance of consistent reference values and assessment methods when comparing micronutrient intake distributions across European countries. Their findings suggested a generally low risk of very low dietary intakes for several vitamins, with vitamin D representing an important exception. Although differences in study populations and dietary methods prevent direct equivalence with the present cohort, these observations provide relevant context for the low LRNI burden identified here alongside the much higher proportion below the vitamin D RNI.

The low LRNI burden was also accompanied by substantial mean intakes of several B vitamins and vitamin C. Mean daily intakes included 2.15 mg of thiamin, 2.43 mg of riboflavin, 58.96 mg NE of niacin, 3.10 mg of vitamin B6 and 181.86 mg of vitamin C. Nevertheless, relatively high cohort-level means should not be taken to demonstrate adequate intake in every participant, particularly when intake distributions are heterogeneous.

Taken together, these results reinforce the importance of interpreting individual vitamin profiles using the appropriate dietary-reference framework rather than combining distinct thresholds into a single classification of micronutrient adequacy. The present study therefore uses explicit descriptions such as “below LRNI”, “below RNI”, “below AR” and “below PRI”, avoiding terminology implying clinical deficiency where this cannot be established from dietary intake data alone.

### 4.5. Food Source Attribution Adds Information Beyond Vitamin Totals

Food source attribution provided one of the most informative extensions of the vitamin profile. Whereas participant-level nutrient totals quantify estimated vitamin intake, source-resolved analysis identifies the foods through which that exposure occurs. The present findings revealed markedly different food-group contributions between vitamins, indicating that overall micronutrient exposure was supported by several distinct dietary pathways rather than a single dominant food pattern.

Vitamin C exhibited the most concentrated plant-food profile. Fruit accounted for 53.4% of reconstructed intake and vegetables for a further 32.9%, together contributing 86.3%. This distribution is consistent with the established importance of fruit and vegetables as major dietary vitamin C sources and with findings from population-based European nutritional surveys [13]. Mean vitamin C intake in the present cohort was 181.86 mg/day, although comparisons with other student populations should consider differences in dietary-assessment methods and portion-size estimation. The strong contribution of fruit and vegetables nevertheless demonstrates that food source patterns provide interpretable information beyond total vitamin intake.

Vitamin B12 showed a contrasting profile, with red and processed meat (27.8%), dairy products and alternatives (27.5%) and fish and seafood (23.9%) providing the principal contributions. Together, these three categories accounted for 79.2% of reconstructed vitamin B12 intake. The predominance of animal-source foods is consistent with European food source studies, including the Hellenic National Nutrition and Health Survey [13]. The distribution also illustrates that vitamin B12 exposure in this cohort was derived from several complementary animal-source food groups rather than meat consumption alone.

The remaining B vitamins exhibited more distributed source profiles. Cereals and grains were the leading contributor to thiamin (28.1%) and also provided important contributions to niacin and vitamin B6. Dairy products and alternatives were the principal riboflavin source (30.3%), while poultry was the leading niacin contributor (25.5%). Vitamin B6 was supplied by cereals and grains (18.5%), poultry (16.9%), red and processed meat (16.7%), vegetables (13.9%) and fruit (12.2%). These patterns demonstrate that B-vitamin exposure was supported by combinations of cereal-based foods, animal-source products and plant foods.

Comparable food source patterns have been reported in the Spanish ANIBES study. Mielgo-Ayuso et al. [40], analysing a representative Spanish population, identified cereals and grains as an important thiamin source (23.9%) and milk and dairy products as the leading contributor to riboflavin (32.2%), values broadly comparable to the contributions of 28.1% and 30.3%, respectively, observed in the present cohort. In ANIBES, meat and meat products were the leading sources of thiamin, niacin and vitamin B6, whereas cereals, vegetables and dairy products also made substantial contributions [40]. These similarities and differences illustrate how commonly consumed food groups can make important contributions to B-vitamin intake across populations, while differences in food-group classification, dietary methodology and consumption patterns limit direct quantitative comparisons.

Vitamin E also showed a distributed food source profile. Vegetables were the largest reconstructed contributor (26.1%), followed by discretionary foods (18.3%), cereals and grains (10.8%), fish and seafood (10.5%) and fruit (8.5%). These results relate to the total vitamin E variable used for dietary exposure and food source attribution and should not be interpreted as a separate characterisation of α-tocopherol intake.

Vitamin A presented a different source distribution, dominated by vegetables (45.9%), which plausibly reflects their contribution to provitamin A carotenoid exposure. Dairy products and alternatives provided a further 11.6%. Notably, soft drinks, energy drinks and sports drinks accounted for 8.8% of the reconstructed vitamin A total. This contribution requires careful interpretation because historical branded-product matching may not fully capture differences in product composition and formulation. However, targeted sensitivity analysis excluding an influential beverage proxy did not alter the dominant vitamin A source or the identity of the five highest-ranked food groups, supporting the stability of the overall source pattern.

An additional methodological strength was that vitamin contributions were reconstructed independently from the original food-level records and CoFID composition data before calibration to the participant-level Nutritics totals. Agreement between the raw CoFID reconstruction and Nutritics was high for most vitamins, and calibration produced minimal changes in food-group ranking. Pearson correlations between raw and calibrated food-group contribution profiles ranged from approximately 0.997 to 1.000, and the same five leading groups were retained before and after calibration for all reconstructed vitamins.

Importantly, the stability of raw and calibrated food source profiles should not be confused with independent agreement between CoFID-derived participant totals and the original Nutritics estimates. These analyses address different aspects of the reconstruction. The lower participant-level concordance observed for vitamin B6 (*r* = 0.826) and the substantially incomplete composition coverage for vitamin K1 illustrate why food source reliability must be assessed separately for each nutrient. Consequently, vitamin K1 source attribution was retained only as an exploratory supplementary analysis rather than presented as directly equivalent to the principal food source findings.

Overall, the source-resolved analysis demonstrates that food-group contributions can provide nutritionally meaningful information not apparent from vitamin totals alone. This may be particularly relevant for the design of food-based dietary strategies because nutritional interventions generally operate through the selection, availability and composition of foods rather than through abstract nutrient quantities.

### 4.6. Sex- and Geographical-Background Differences

Sex-related variation was relatively limited after adjustment for total energy intake and correction for multiple testing. Vitamin A was the only vitamin showing an FDR-significant adjusted sex difference, with males having lower intake than females (−0.484 SD; *q* = 0.029). Although nominal differences were observed for several additional vitamins, including vitamins E, K1 and B6, these associations did not remain significant after BH-FDR correction. The findings therefore indicate a nutrient-specific sex-related pattern rather than a consistent difference across the vitamin panel.

Previous population-based studies have also identified sex-related variation in micronutrient intake, although its magnitude and direction depend on the nutrients examined, dietary-assessment methods, energy intake and population characteristics. Derbyshire [35], using UK National Diet and Nutrition Survey data, reported differences in micronutrient exposure across demographic groups, while Rippin et al. [37] documented variation in adult nutrient intake by sex and country across European national dietary surveys. These findings provide a broader context for the present results but do not imply that the same sex-related patterns should be reproduced in every young adult population. In particular, the restricted age range and energy-adjusted statistical framework used here support the interpretation of the observed vitamin A difference as a specific within-cohort association.

Geographical-background-related variation was more extensive. Global FDR-significant differences were identified for vitamins E and K1, niacin, vitamin B6 and vitamin B12 in the primary analyses. Pairwise comparisons indicated higher vitamin E intake in participants from Asia than in those from Africa, and higher vitamin K1 intake in those from Europe than in those from Africa. Niacin and vitamin B6 intakes were lower in participants from Asia than in one or both comparison groups, while vitamin B12 intake was lower in the Asian than in the African group.

International nutritional studies provide evidence that micronutrient intake may differ across university populations and geographical settings. Dalibalta et al. [41], comparing university students from the United Arab Emirates and the United Kingdom, identified differences in dietary and nutrient intake between the two study populations. More broadly, comparative studies of adults from different geographical or ancestral backgrounds have documented variation in food-group and nutrient consumption [42]. These findings support the relevance of examining dietary heterogeneity within geographically diverse populations, although the demographic definitions and study settings differ from those used in the present investigation.

Importantly, these comparisons do not establish the underlying reasons for the associations identified in the DMU cohort. Geographical background was derived from region of birth and was used as a broad demographic descriptor rather than a direct measure of ethnicity, cultural identity, genetic ancestry, socioeconomic circumstances or dietary acculturation. Participants within each geographical category may have substantially different food preferences, living arrangements, financial circumstances and previous dietary experiences. The observed associations should therefore be interpreted as evidence of dietary heterogeneity within the cohort rather than as inherent nutritional characteristics of particular geographical groups.

The sensitivity analyses provide an important qualification to the demographic findings. Global geographical-background differences in vitamins E, K1, niacin, and B6 remained FDR-significant under additional demographic adjustment and HC3 robust inference, whereas vitamin B12 did not. The separate exclusion of five high-energy-intake observations further supported the stability of the vitamin A sex association and the global geographical associations for vitamins E, K1, and B6. However, the global associations for niacin and vitamin B12 no longer met the BH-FDR significance threshold, and none of the seven originally significant geographical pairwise contrasts retained FDR significance. These results indicate that the evidence for some specific geographical differences depends on the inclusion of high-energy-intake observations. Accordingly, the primary geographical findings should be interpreted cautiously, particularly at the pairwise-comparison level.

Together, these results highlight the importance of examining demographic variation using nutrient-specific models rather than assuming that sex or geographical background exerts uniform effects across the vitamin panel. The findings also suggest that future investigations would benefit from a more detailed characterisation of individual dietary practices and socioeconomic circumstances to clarify the factors associated with variation in micronutrient intake.

### 4.7. Vitamin E and Vitamin K1 Illustrate Why Nutrient Definition Matters

Vitamin E provides an important methodological example. The validated dataset contained a total vitamin E measure and a separate historical α-tocopherol field. The EFSA AI, however, is defined specifically in relation to α-tocopherol [25].

Assigning the total vitamin E variable to the α-tocopherol AI would therefore create a mismatch between measured exposure and reference definition. Conversely, the available information was insufficient to establish that the historical α-tocopherol field represented complete dietary α-tocopherol exposure comparable with the EFSA AI. The decision not to classify participants against this benchmark was therefore intentionally conservative.

Vitamin K1 illustrates a different issue. Although participant-level Nutritics K1 estimates were available for all 111 participants, the completeness of the underlying food-level vitamin K1 composition data could not be independently verified from the archived records. Furthermore, usable CoFID composition was available for only 47.7% of cohort food grams, and the median absolute relative difference between the independent CoFID reconstruction and Nutritics estimates was 23.2%. Consequently, vitamin K1 dietary-reference comparisons and demographic associations, including the Europe–Africa contrast, are interpreted as exploratory. Food source attribution was likewise retained exclusively as an exploratory supplementary analysis.

These examples reinforce a wider principle in nutritional epidemiology: the availability of a numerical variable does not in itself justify every possible comparison. Analytical definitions, food-composition coverage, and the exact reference criterion must all support the intended interpretation.

### 4.8. Strengths, Limitations, and Priorities for Future Research

Several characteristics strengthen the present analysis. The narrow 18–22-year age range provides a focused assessment of emerging adulthood, while the geographically heterogeneous cohort permitted examination of dietary variation within a common university environment.

The dietary data were reconstructed and quality-controlled from the original participant-level records rather than inferred retrospectively from published summary statistics. Weekly-to-daily conversion was explicitly verified, and the same validated participant-level nutrient dataset was used for descriptive, dietary-reference, and inferential analyses.

The dietary-reference framework was deliberately nutrient-specific. Sex-, energy-, protein-, and body-weight-dependent criteria were implemented where required, and LRNI, RNI, AR, PRI, AI, and safe-intake values were not treated as interchangeable. The decision not to classify vitamin E against an α-tocopherol AI when analytical equivalence could not be established represents an additional methodological safeguard.

The statistical approach incorporated standardisation, total-energy adjustment, a predefined multiplicity framework, and fully adjusted HC3 sensitivity analyses. Interpretation was based on effect estimates, confidence intervals, and FDR-adjusted q-values rather than nominal *p*-values alone.

The independent food source reconstruction represents a further strength. Source contributions were generated from original food-level consumption records and an external UK composition system, quantitatively checked against the corresponding Nutritics totals, and evaluated for structural stability before and after calibration. Nutrient-specific limitations in reconstruction quality were retained rather than concealed; most notably, vitamin K1 food source attribution was kept exploratory because of incomplete composition coverage.

These strengths should be interpreted alongside several limitations. The study comprised 111 volunteers from a single English university and was predominantly female. It was not designed to provide nationally representative prevalence estimates, and subgroup analyses have limited precision.

Dietary intake was assessed using an expanded semi-quantitative FFQ based on the extensively evaluated EPIC-Norfolk instrument, whose original methodology has been validated using dietary records and nutritional biomarkers [19,20]. The adaptations introduced for the present geographically diverse university population, including additional food items and the study-specific dietary-assessment approach, were not independently revalidated against a reference method. Consequently, some uncertainty remains in absolute nutrient-intake estimates, particularly when interpreting dietary-reference comparisons [43]. Nevertheless, the same standardised assessment procedure was applied across participants, providing a consistent basis for within-cohort comparisons. As separately reported vitamin and mineral supplement contributions were not quantified, the dietary-reference comparisons reflect recorded food-based vitamin intake rather than total intake from foods and supplements combined.

The analyses characterise dietary intake rather than biochemical vitamin status. No participant should therefore be classified as clinically vitamin deficient on the basis of these results. This distinction is especially important for vitamin D, for which circulating status depends on determinants beyond dietary intake.

Residual confounding may affect demographic comparisons. Although total energy intake was incorporated in the primary models and age and complementary demographic factors were included in sensitivity analyses, socioeconomic position, living arrangements, food security, cooking facilities, migration history, and other behavioural factors could not be comprehensively represented.

Geographical background was based on region of birth and should not be interpreted as a surrogate for ethnicity, genetic ancestry, or culture. The broad categories necessarily conceal substantial within-group heterogeneity.

The dietary data provide a defined historical snapshot of vitamin intake and food source patterns among university students assessed in early 2016. Since then, the UK food environment has evolved, including changes in food availability, purchasing conditions, and product formulation. More recent UK dietary surveillance has documented the influence of the COVID-19 pandemic and rising food prices on food availability and purchasing behaviours [32], while vitamin D fortification has remained an area of national nutritional policy assessment [31]. Importantly, methodological changes introduced into the National Diet and Nutrition Survey in 2019 prevent direct numerical comparisons of its more recent findings with earlier survey periods [32]. The present results should therefore be understood within their original dietary context rather than as estimates of contemporary university student intake. Nevertheless, the concentrated January-to-mid-March assessment period and the consistent analytical framework provide a historical reference for future studies employing comparable methodologies.

Future research should prioritise larger multi-university cohorts with repeated dietary assessments across emerging adulthood. Combining validated repeated dietary methods with micronutrient biomarkers would be particularly valuable for distinguishing dietary reference-value profiles from biochemical status.

Prospective studies should also capture socioeconomic circumstances, food security, living arrangements, cooking facilities, physical activity, supplement use where relevant, and detailed migration or acculturation variables. Such data would permit a more informative investigation of demographic heterogeneity than broad geographical categories alone.

Finally, repeated FoodName-level or product-level assessment would allow future studies to quantify the impact of changes in fortification, reformulation, and the university food environment on vitamin exposure and its dietary sources.

## 5. Conclusions

In this geographically diverse cohort of 111 university students aged 18–22 years, dietary vitamin exposure was characterised by nutrient-specific rather than generalised reference-value shortfalls.

Intakes below UK LRNIs were uncommon across vitamins A, B1, B2, B3, B6, B12, and C, with 93.7% of participants below none of the seven prespecified LRNIs. This finding should not be interpreted as evidence of universal vitamin adequacy because the LRNI describes the extreme lower end of the requirement distribution rather than an optimal dietary target.

In contrast, 94.6% of participants had dietary vitamin D intake below the UK RNI, while 27.9% and 48.6% had folate intake below the EFSA AR and PRI, respectively. These comparisons describe dietary exposure and do not establish biochemical deficiency.

Food source attribution identified distinct dietary pathways underlying the vitamin profile. Fish/seafood was the leading source of vitamin D; meat, dairy/alternatives, and fish supplied most vitamin B12; vegetables, cereals/grains, and fruit were major folate sources; and fruit and vegetables dominated vitamin C intake.

Adjusted sex-related differences were limited principally to vitamin A, whereas several vitamins showed variation across broad geographical-background groups. These demographic findings should be interpreted as evidence of within-cohort dietary heterogeneity rather than intrinsic characteristics of geographical groups.

Overall, integrating nutrient-specific reference values with independently reconstructed food sources provided a more informative characterisation of vitamin exposure than average intake alone. This approach may help identify both the nutrients and the dietary pathways most relevant to future nutritional surveillance and intervention during emerging adulthood.

## Figures and Tables

**Table 1:** Distribution of daily vitamin intakes in the study cohort (*N* = 111).

Vitamin	Unit	*N*	Mean ± SD	Median [Q1–Q3]	Range	P5–P95
**Vitamin A (total RE)**	µg/day	111	1013.74 ± 739.81	875.47 [561.72–1210.53]	157.04–5397.68	307.41–2401.55
**Vitamin D**	µg/day	111	3.96 ± 3.13	3.06 [1.78–5.21]	0.05–18.15	0.78–9.94
**Vitamin E**	mg/day	111	11.58 ± 6.49	10.49 [7.04–13.25]	1.22–34.00	4.35–25.47
**Vitamin K1 (phylloquinone)**	µg/day	111	102.18 ± 95.29	83.12 [43.51–122.25]	3.39–702.10	14.91–280.64
**Thiamin (B1)**	mg/day	111	2.15 ± 1.08	1.90 [1.38–2.58]	0.77–6.76	0.93–4.26
**Riboflavin (B2)**	mg/day	111	2.43 ± 1.43	1.96 [1.48–2.94]	0.46–8.34	0.99–5.35
**Niacin (B3, total NE)**	mg/day	111	58.96 ± 30.65	52.30 [38.18–68.82]	21.01–185.41	24.62–117.42
**Vitamin B6**	mg/day	111	3.10 ± 1.73	2.70 [1.95–4.05]	0.94–10.13	1.18–6.23
**Folate (B9, total DFE)**	µg/day	111	387.74 ± 208.50	341.71 [227.65–499.46]	92.64–1080.04	158.11–767.68
**Vitamin B12**	µg/day	111	9.57 ± 5.90	8.09 [5.51–12.56]	0.50–35.81	3.37–20.13
**Vitamin C**	mg/day	111	181.86 ± 132.25	154.93 [91.69–227.28]	29.10–706.48	40.32–443.57

RE, retinol equivalent; NE, niacin equivalent; DFE, dietary folate equivalent. Values are presented as daily dietary intakes.

**Table 2:** Summary of vitamin-specific dietary reference values and the proportion of participants below each benchmark.

Vitamin	Reference Benchmark	Reference Type	Reference Value/Rule	Below Benchmark, *n*/*N*	Below Benchmark, % (95% CI)
**Vitamin A (total RE)**	UK LRNI	LRNI	Men 300 µg RE/day; women 250 µg RE/day	5/111	4.5 (1.9–10.1)
**Thiamin (B1)**	UK LRNI	LRNI	0.23 mg/1000 kcal × participant energy	0/111	0.0 (0.0–3.3)
**Riboflavin (B2)**	UK LRNI	LRNI	0.8 mg/day	1/111	0.9 (0.2–4.9)
**Niacin (B3, total NE)**	UK LRNI	LRNI	4.4 mg NE/1000 kcal × participant energy	0/111	0.0 (0.0–3.3)
**Vitamin B6**	UK LRNI	LRNI	11 µg/g dietary protein × participant protein	0/111	0.0 (0.0–3.3)
**Vitamin B12**	UK LRNI	LRNI	1.0 µg/day	1/111	0.9 (0.2–4.9)
**Vitamin C**	UK LRNI	LRNI	10 mg/day	0/111	0.0 (0.0–3.3)
**Vitamin D**	UK RNI	RNI	10 µg/day	105/111	94.6 (88.7–97.5)
**Folate (B9, total DFE)**	EFSA AR	AR	250 µg DFE/day	31/111	27.9 (20.4–36.9)
**Folate (B9, total DFE)**	EFSA PRI	PRI	330 µg DFE/day	54/111	48.6 (39.6–57.8)
**Vitamin E**	EFSA AI	AI	α-tocopherol: men 13 mg/day; women 11 mg/day	NOT CLASSIFIED	—
**Vitamin K1 (phylloquinone)**	UK safe intake	safe intake	~1 µg/kg body weight/day × participant weight	44/111	39.6 (31.0–48.9)
**Vitamin K1 (phylloquinone)**	EFSA AI	AI	70 µg/day	47/111	42.3 (33.6–51.6)

LRNI, lower reference nutrient intake; RNI, reference nutrient intake; AR, average requirement; PRI, population reference intake; AI, adequate intake. Reference types are not directly interchangeable. Comparisons with AI or safe-intake values are descriptive and should not be interpreted as estimates of individual inadequacy. Vitamin E is not classified against the EFSA AI because equivalence and completeness of the historical alpha-tocopherol field could not be established. Participant-specific thresholds were used for thiamin and niacin (energy), vitamin B6 (protein), vitamin K1 UK safe intake (body weight), and vitamin A (sex-specific values).

**Table 3:** Primary adjusted comparisons of vitamin intakes by sex and geographical background.

Vitamin	Sex Comparisons (Male–Female; *N* = 111)				Geographical Background (Global Test; *N* = 109)			
	Adj. Δ (SD)	95% CI	*p*	BH-FDR *q*	*F*	Partial *η*^2^	*p*	BH-FDR *q*
Vitamin A (total RE)	−0.484	−0.796 to −0.172	0.003	**0.029**	1.953	0.036	0.147	0.202
Vitamin D	−0.087	−0.463 to 0.289	0.648	0.891	2.626	0.048	0.077	0.121
Vitamin E	−0.280	−0.533 to −0.028	0.030	0.083	4.828	0.084	0.010	**0.034**
Vitamin K1 (phylloquinone)	−0.468	−0.825 to −0.112	0.011	0.058	4.575	0.080	0.012	**0.034**
Thiamin (B1)	0.109	−0.069 to 0.287	0.226	0.415	0.981	0.018	0.378	0.416
Riboflavin (B2)	−0.084	−0.344 to 0.176	0.523	0.823	3.148	0.057	0.047	0.086
Niacin (B3, total NE)	0.190	−0.050 to 0.430	0.119	0.263	5.023	0.087	0.008	**0.034**
Vitamin B6	0.328	0.053 to 0.603	0.020	0.072	5.594	0.096	0.005	**0.034**
Folate (B9, total DFE)	0.011	−0.270 to 0.292	0.938	0.938	1.400	0.026	0.251	0.307
Vitamin B12	−0.024	−0.357 to 0.308	0.885	0.938	3.952	0.070	0.022	**0.049**
Vitamin C	−0.013	−0.335 to 0.308	0.934	0.938	0.425	0.008	0.655	0.655

Primary sex and geographical-background models were adjusted for total energy intake. Sex comparisons report adjusted male–female differences in standard deviation (SD) units (*N* = 111). Geographical-background tests compare Africa, Asia and Europe (*N* = 109); the Other group (*n* = 2) was excluded from formal inference. Multiple testing was controlled using the Benjamini–Hochberg false-discovery rate (BH-FDR) within the respective prespecified testing families. Fully adjusted HC3 sensitivity models are reported in the Supplementary Material. Bold values indicate statistically significant results after BH-FDR correction (*q* < 0.05).

**Table 4:** Top five food-group contributors to vitamin intake based on the calibrated CoFID reconstruction.

Vitamin	1st Contributor	2nd	3rd	4th	5th	Top 5 Cumulative
**Vitamin A (total RE)**	Vegetables (45.9%)	Dairy/Alternatives (11.6%)	Soft/energy/sports drinks (8.8%)	Cereals/Grains (7.6%)	Mixed dishes/Condiments (7.1%)	81.0%
**Vitamin D**	Fish/Seafood (31.8%)	Cereals/Grains (19.1%)	Red/processed meat (16.7%)	Eggs (13.5%)	Dairy/Alternatives (6.0%)	87.2%
**Vitamin E**	Vegetables (26.1%)	Discretionary foods (18.3%)	Cereals/Grains (10.8%)	Fish/Seafood (10.5%)	Fruit (8.5%)	74.2%
**Thiamin (B1)**	Cereals/Grains (28.1%)	Vegetables (19.6%)	Fruit (13.3%)	Red/processed meat (11.1%)	Dairy/Alternatives (5.7%)	77.9%
**Riboflavin (B2)**	Dairy/Alternatives (30.3%)	Cereals/Grains (20.9%)	Red/processed meat (11.0%)	Vegetables (7.6%)	Discretionary foods (6.3%)	76.2%
**Niacin (B3, total NE)**	Poultry (25.5%)	Cereals/Grains (21.1%)	Red/processed meat (20.1%)	Fish/Seafood (8.8%)	Vegetables (6.1%)	81.6%
**Vitamin B6**	Cereals/Grains (18.5%)	Poultry (16.9%)	Red/processed meat (16.7%)	Vegetables (13.9%)	Fruit (12.2%)	78.2%
**Folate (CoFID total)**	Vegetables (31.0%)	Cereals/Grains (18.3%)	Fruit (16.7%)	Dairy/Alternatives (9.4%)	Pulses (6.1%)	81.6%
**Vitamin B12**	Red/processed meat (27.8%)	Dairy/Alternatives (27.5%)	Fish/Seafood (23.9%)	Cereals/Grains (9.5%)	Discretionary foods (4.3%)	93.0%
**Vitamin C**	Fruit (53.4%)	Vegetables (32.9%)	Soft/energy/sports drinks (3.6%)	Dairy/Alternatives (2.7%)	Pulses (2.1%)	94.8%

Values in parentheses are calibrated percentage contributions. Folate refers to CoFID total folate for food source attribution, whereas total DFE is retained for dietary-reference analyses. Vitamin K1 food source attribution is presented separately in Supplementary Table S13 because usable CoFID composition covered approximately 47.7% of cohort food grams.

## Data Availability

Aggregated data supporting the main tables and figures, supplementary analytical outputs, figure-source data, cohort-aggregated FoodName-level vitamin source attribution data, vitamin-specific R analysis code, and methodological and quality-assurance documentation are available in Figshare at https://doi.org/10.6084/m9.figshare.34330503 [44]. Original participant-level dietary records, FFQ/Nutritics/Access source files, participant-linked food-intake data, individual dietary-reference classifications, and complete third-party food-composition datasets are not publicly available owing to ethical, institutional, data-protection, and redistribution restrictions. Requests for additional de-identified participant-level information may be considered by the corresponding author, subject to applicable ethical and institutional approvals.

## Supplementary Materials

The following supporting information can be downloaded at https://www.mdpi.com/article/10.3390/foods15213997/s1: Table S1. Study context: related investigations and characteristics of the historical university cohort. Panel A. Scope of related analyses and outcomes addressed in the present vitamin-intake study. Panel B. Demographic, anthropometric, and dietary characteristics of participants by geographical background; Table S2. Daily vitamin intakes by sex; Table S3. Daily vitamin intakes by geographical background; Table S4. Primary adjusted sex comparisons across all vitamins; Table S5. Primary global geographical-background tests across all vitamins; Table S6. Primary pairwise geographical-background comparisons across all vitamins; Table S7. Fully adjusted HC3 sensitivity analysis for sex comparisons; Table S8. Fully adjusted HC3 sensitivity analysis for global geographical-background tests; Table S9. Fully adjusted HC3 sensitivity analysis for pairwise geographical-background comparisons; Table S10. Full dietary-reference comparison table; Table S11. Agreement between raw CoFID-reconstructed vitamin totals and original Nutritics totals at participant level; Table S12. Concordance of food-group source structure before and after calibration; Table S13. Exploratory top-five food-group contributors to vitamin K1; Figure S1. Distribution of the number of prespecified vitamins below the UK LRNI per participant. The count includes vitamin A, thiamin (B1), riboflavin (B2), niacin (B3), vitamin B6, vitamin B12 and vitamin C only; Figure S2. Complete forest plot of primary adjusted sex and pairwise geographical-background comparisons across the vitamin panel. Estimates are standardized differences with 95% confidence intervals; Figure S3. Complete forest plot of fully adjusted HC3 sensitivity analyses across the vitamin panel. Estimates are standardized differences with 95% confidence intervals; Figure S4. Participant-level comparison of raw CoFID-reconstructed vitamin intakes with the corresponding original Nutritics totals. The dashed line indicates equality between the two estimates. For folate, the plotted comparison is between CoFID total folate (FOLT) and Nutritics natural folate; these measures are not directly equivalent.
