# Supplementary material for: Dietary Vitamin Intakes, Reference-Value Profiles, and Major Food Sources in a Historical Cohort of Geographically Diverse UK University Students
Source: Foods. 2026 Nov 7;15(21):3997. doi: 10.3390/foods15213997 (PMC13762913; doi:10.3390/foods15213997)
Supplement: Supplementary file 1 [file foods-15-03997-s001.zip › Supplementary Figs vits art_revised.pdf]

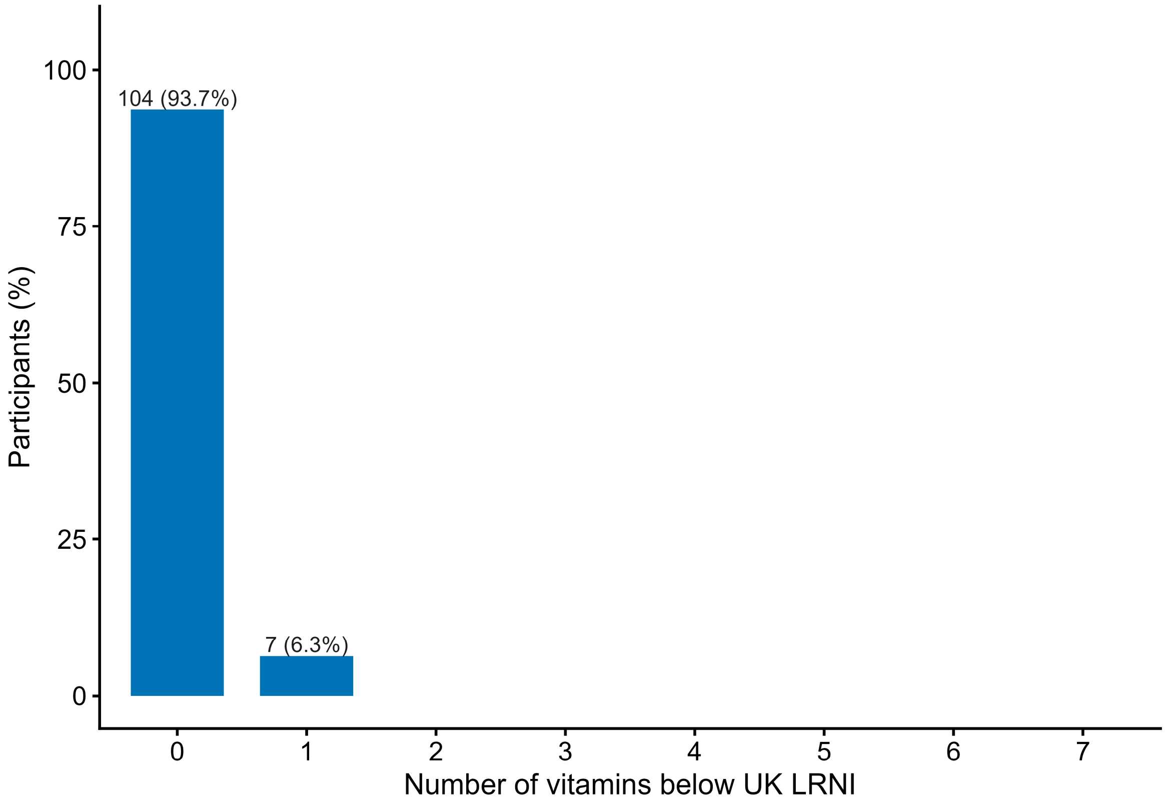

Figure S1. Distribution of the number of prespecified vitamins below the UK LRNI per participant. The count includes vitamin A, thiamin (B1), riboflavin (B2), niacin (B3), vitamin B6, vitamin B12 and vitamin C only.

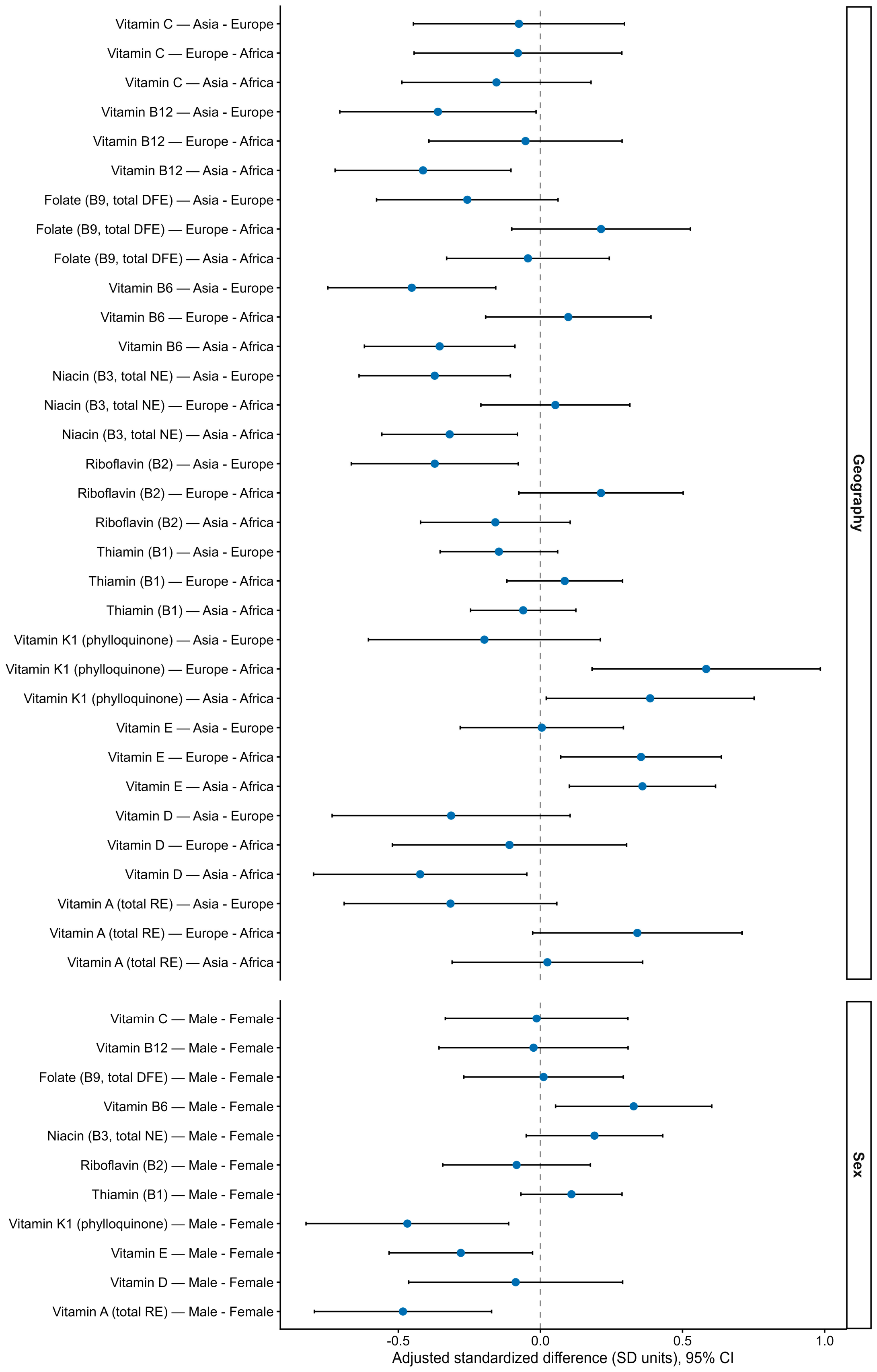

Figure S2. Complete forest plot of primary adjusted sex and pairwise geographical-background comparisons across the vitamin panel. Estimates are standardized differences with 95% confidence intervals.

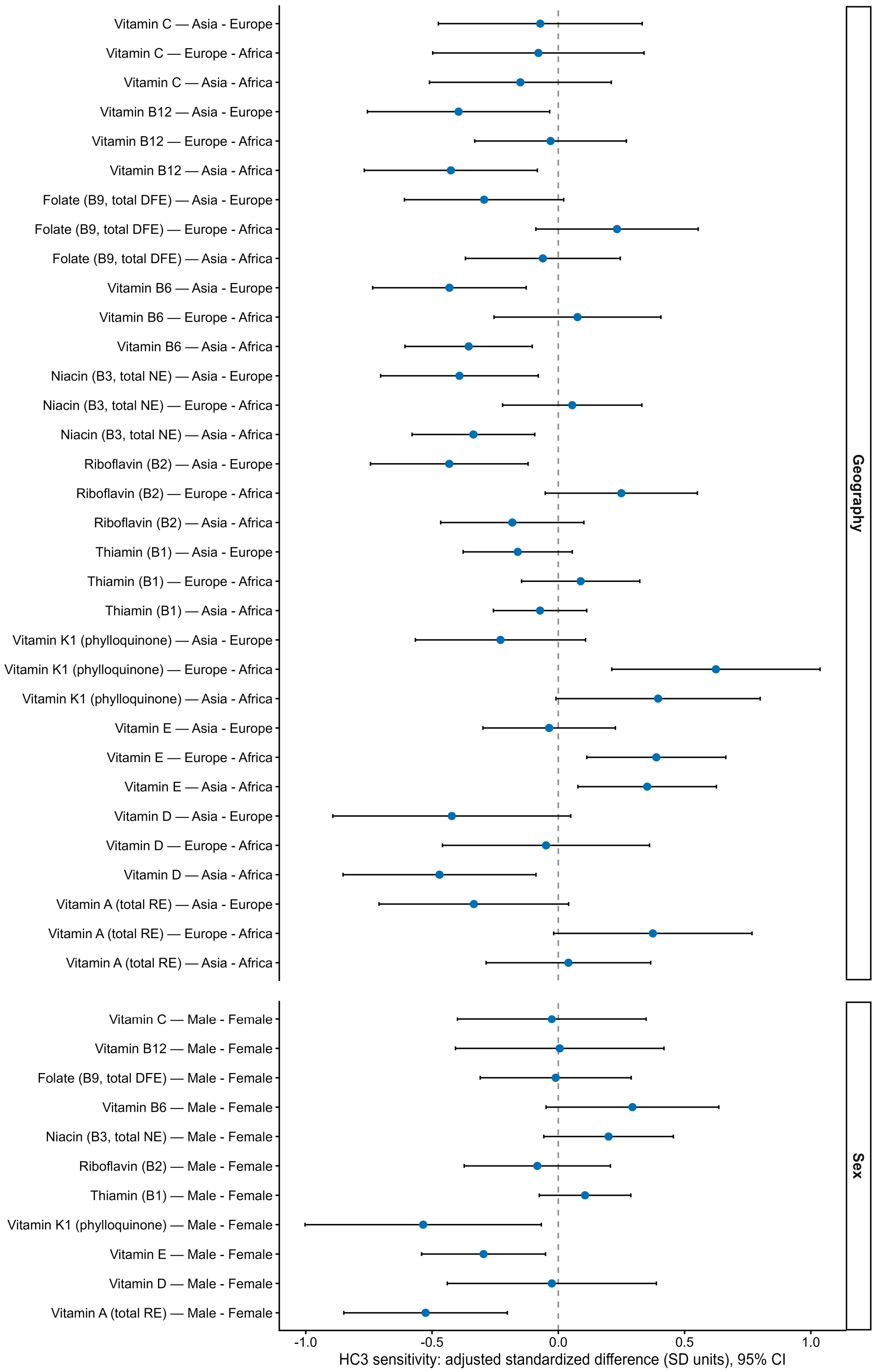

Figure S3. Complete forest plot of fully adjusted HC3 sensitivity analyses across the vitamin panel. Estimates are standardized differences with 95% confidence intervals.

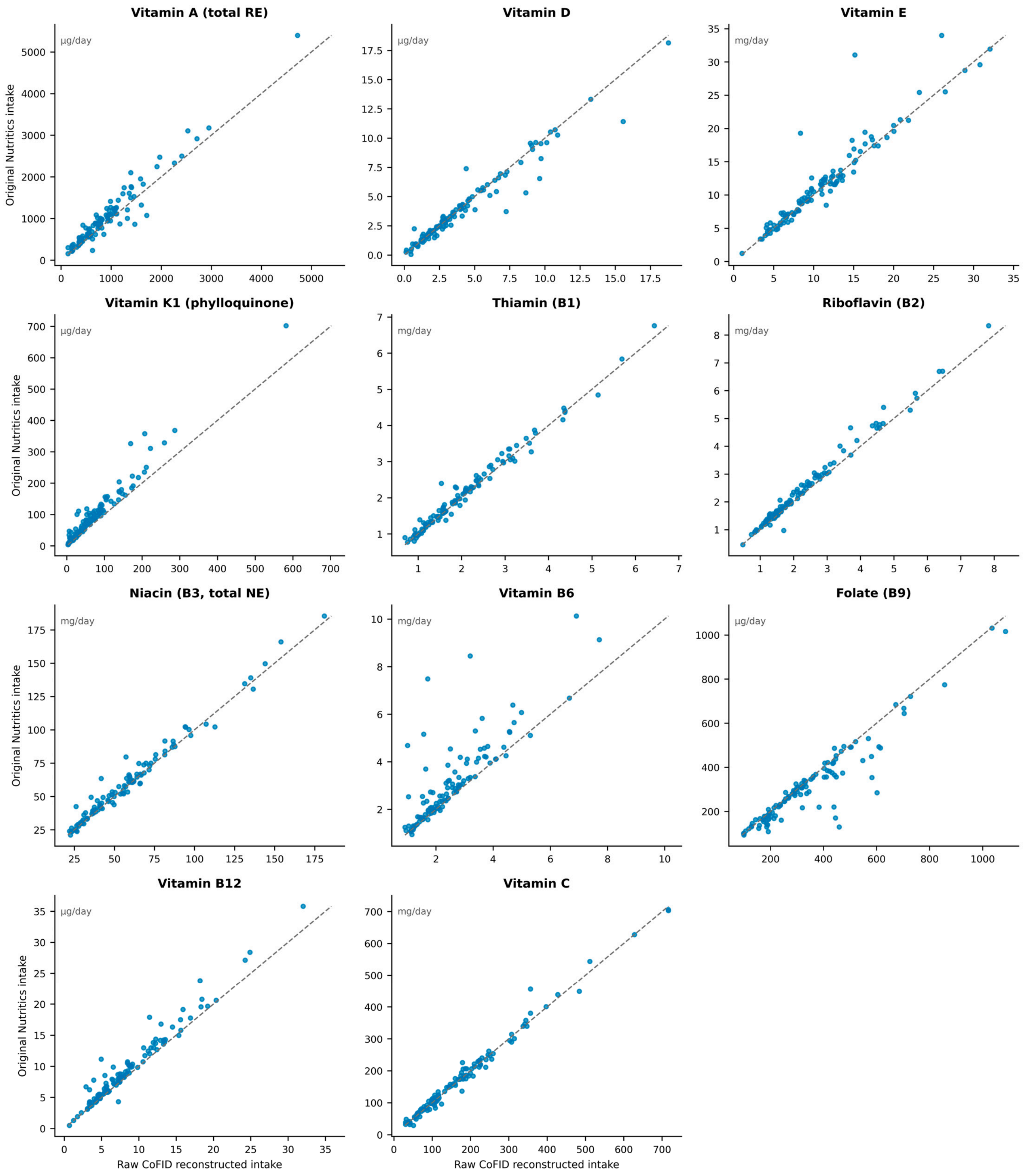

Figure S4. Participant-level comparison of raw CoFID-reconstructed vitamin intakes with the corresponding original Nutritics totals. The dashed line indicates equality between the two estimates. For folate, the plotted comparison is between CoFID total folate (FOLT) and Nutritics natural folate; these measures are not directly equivalent.
