# Supplementary material for: Dietary Vitamin Intakes, Reference-Value Profiles, and Major Food Sources in a Historical Cohort of Geographically Diverse UK University Students
Source: Foods. 2026 Nov 7;15(21):3997. doi: 10.3390/foods15213997 (PMC13762913; doi:10.3390/foods15213997)
Supplement: Supplementary file 1 [file foods-15-03997-s001.zip › Supplementary Tables vits art_revised.pdf]

Table S1. Study context: related investigations and characteristics of the historical university cohort.

Panel A. Scope of related analyses and outcomes addressed in the present vitamin-intake study.

Publication	Main research question	Principal findings or outcomes reported
Peña-Fernández et al., 2023 [14]	Preliminary dietary vitamin D intake	Initial estimates of vitamin D intake and comparisons by sex and geographical background.
Peña-Fernández et al., 2026 [15]	Whole-diet patterns and adiposity	Associations of three PCA-derived dietary-pattern scores with BMI, body fat percentage and visceral-fat index; no primary association survived BH-FDR correction.
Peña-Fernández et al., 2026 [16]	Food-group profiles and dietary patterns	Food-group consumption and three PCA-derived dietary patterns, including adjusted differences by sex and geographical background.
Present study	Vitamin-specific dietary exposure, reference values and sources	Distributions of 11 vitamins; UK and EFSA reference-value profiles; adjusted vitamin-level demographic comparisons; CoFID-based reconstruction of vitamin-specific food-group sources.

The studies share the same historical cohort but address different research questions and outcome definitions. BH-FDR, Benjamini–Hochberg false-discovery-rate correction; BMI, body mass index; PCA, principal component analysis.

Panel B. Demographic, anthropometric, and dietary characteristics of participants by geographical background.

Characteristic	Overall (N = 111)	Africa (n = 41)	Asia (n = 41)	Europe (n = 27)	Other (n = 2)
Female, n (%)	78 (70.3)	31 (75.6)	28 (68.3)	18 (66.7)	1 (50.0)
Male, n (%)	33 (29.7)	10 (24.4)	13 (31.7)	9 (33.3)	1 (50.0)
Age (years)	20.43 ± 1.14	20.39 ± 0.86	20.73 ± 1.28	20.04 ± 1.22	20.50 ± 0.71
Energy intake (kcal/day)	2524.10 ± 1256.09	2300.29 ± 1008.37	2874.20 ± 1460.50	2365.01 ± 1224.96	2082.92 ± 627.36
Protein intake (g/day)	118.29 ± 62.31	113.07 ± 56.91	122.64 ± 61.68	121.68 ± 73.43	90.17 ± 37.41
Body weight (kg)	66.43 ± 13.49	66.23 ± 10.34	62.74 ± 15.63	72.21 ± 13.17	68.30 ± 0.57
Missing observations, n	0	0	0	0	0

Values are presented as mean ± standard deviation (SD) for continuous variables and n (%) for categorical variables. Sex percentages were calculated within geographical-background groups. The categories reflect the corrected frozen analytical dataset (N = 111). No values were missing for the variables listed. The two participants in the Other category were retained in descriptive analyses but excluded from geographical-background inference (N = 109); statistics for this category are descriptive only.

Table S2. Daily vitamin intakes by sex.

Vitamin	Unit	Female *N*	Female mean ± SD	Female median [Q1–Q3]	Male *N*	Male mean ± SD	Male median [Q1–Q3]
**Vitamin A (total RE)**	µg/day	78	1024.71 ± 801.10	876.12 [549.18–1116.90]	33	987.83 ± 579.79	861.87 [611.35–1243.46]
**Vitamin D**	µg/day	78	3.80 ± 3.27	2.91 [1.66–4.70]	33	4.34 ± 2.78	3.60 [2.42–6.39]
**Vitamin E**	mg/day	78	11.10 ± 6.48	9.48 [6.84–12.82]	33	12.69 ± 6.48	11.84 [9.20–16.53]
**Vitamin K1 (phylloquinone)**	µg/day	78	97.21 ± 76.90	82.16 [45.69–115.41]	33	113.92 ± 129.56	86.25 [34.46–146.59]
**Thiamin (B1)**	mg/day	78	1.94 ± 0.95	1.61 [1.32–2.28]	33	2.65 ± 1.20	2.40 [1.94–3.05]
**Riboflavin (B2)**	mg/day	78	2.27 ± 1.40	1.76 [1.41–2.57]	33	2.81 ± 1.45	2.59 [1.70–3.05]
**Niacin (B3, total NE)**	mg/day	78	52.97 ± 26.81	45.25 [33.30–62.94]	33	73.12 ± 34.73	64.76 [55.05–81.42]
**Vitamin B6**	mg/day	78	2.68 ± 1.31	2.34 [1.68–3.18]	33	4.08 ± 2.17	3.38 [2.71–4.57]
**Folate (B9, total DFE)**	µg/day	78	360.40 ± 203.93	300.17 [211.61–456.50]	33	452.34 ± 207.93	436.41 [310.48–543.84]
**Vitamin B12**	µg/day	78	8.83 ± 5.42	7.64 [5.27–10.68]	33	11.33 ± 6.67	9.94 [7.28–14.37]
**Vitamin C**	mg/day	78	164.77 ± 123.41	125.21 [81.52–210.43]	33	222.24 ± 145.21	186.18 [124.11–261.61]

Values are descriptive statistics for female (*n*=78) and male (*n*=33) participants. SD, standard deviation; Q1–Q3, interquartile range.

Table S3. Daily vitamin intakes by geographical background.

Vitamin	Unit	Asia mean ± SD	Asia median [Q1–Q3]	Africa mean ± SD	Africa median [Q1–Q3]	Europe mean ± SD	Europe median [Q1–Q3]
**Vitamin A (total RE)**	µg/day	1051.68 ± 616.35	891.47 [616.02–1263.20]	968.71 ± 948.21	841.42 [464.82–1092.35]	1047.97 ± 577.11	914.19 [596.44–1229.94]
**Vitamin D**	µg/day	3.71 ± 2.67	3.28 [1.97–4.22]	4.11 ± 2.98	2.97 [2.02–5.10]	4.20 ± 4.00	2.82 [1.60–5.15]
**Vitamin E**	mg/day	13.63 ± 6.56	12.18 [9.24–16.91]	9.60 ± 6.09	7.68 [5.52–11.67]	11.57 ± 6.47	9.39 [6.42–13.87]
**Vitamin K1 (phylloquinone)**	µg/day	115.26 ± 85.80	86.90 [52.28–157.32]	84.34 ± 115.01	56.77 [26.05–100.78]	106.38 ± 76.29	95.84 [51.09–120.10]
**Thiamin (B1)**	mg/day	2.34 ± 1.11	2.19 [1.62–2.86]	1.98 ± 0.97	1.58 [1.32–2.40]	2.12 ± 1.18	1.92 [1.21–2.56]
**Riboflavin (B2)**	mg/day	2.52 ± 1.37	2.24 [1.47–2.91]	2.26 ± 1.44	1.72 [1.52–2.45]	2.56 ± 1.53	2.06 [1.48–2.97]
**Niacin (B3, total NE)**	mg/day	59.85 ± 30.59	57.27 [38.51–74.97]	56.83 ± 27.28	51.85 [39.86–63.52]	61.36 ± 36.26	49.38 [37.18–70.70]
**Vitamin B6**	mg/day	3.04 ± 1.65	2.56 [1.87–3.98]	2.96 ± 1.47	2.56 [1.97–3.69]	3.25 ± 1.97	2.91 [1.99–4.20]
**Folate (B9, total DFE)**	µg/day	411.58 ± 209.40	359.19 [268.04–496.08]	356.82 ± 216.42	286.00 [217.78–453.94]	391.77 ± 202.73	348.49 [243.11–524.00]
**Vitamin B12**	µg/day	9.31 ± 5.46	8.23 [5.52–10.72]	9.73 ± 5.94	8.72 [5.99–11.73]	9.76 ± 6.41	7.14 [5.04–13.34]
**Vitamin C**	mg/day	196.82 ± 147.68	159.11 [115.48–234.94]	178.32 ± 138.36	110.67 [83.38–212.73]	162.02 ± 94.56	160.63 [89.84–227.49]

Values are descriptive statistics for participants classified as Asia (n=41), Africa (n=41) and Europe (n=27). The two participants classified as Other are not included in these formal geographical-background summaries.

Table S4. Primary adjusted sex comparisons across all vitamins.

Vitamin	Contrast	*N*	Adjusted difference (SD)	95% CI	*p*	BH-FDR *q*
**Vitamin A (total RE)**	Male - Female	111	-0.484	-0.796 to -0.172	0.003	0.029
**Vitamin D**	Male - Female	111	-0.087	-0.463 to 0.289	0.648	0.891
**Vitamin E**	Male - Female	111	-0.280	-0.533 to -0.028	0.030	0.083
**Vitamin K1 (phylloquinone)**	Male - Female	111	-0.468	-0.825 to -0.112	0.011	0.058
**Thiamin (B1)**	Male - Female	111	0.109	-0.069 to 0.287	0.226	0.415
**Riboflavin (B2)**	Male - Female	111	-0.084	-0.344 to 0.176	0.523	0.823
**Niacin (B3, total NE)**	Male - Female	111	0.190	-0.050 to 0.430	0.119	0.263
**Vitamin B6**	Male - Female	111	0.328	0.053 to 0.603	0.020	0.072
**Folate (B9, total DFE)**	Male - Female	111	0.011	-0.270 to 0.292	0.938	0.938
**Vitamin B12**	Male - Female	111	-0.024	-0.357 to 0.308	0.885	0.938
**Vitamin C**	Male - Female	111	-0.013	-0.335 to 0.308	0.934	0.938

Primary models were adjusted for total energy intake. Multiple testing was controlled using the Benjamini–Hochberg false-discovery rate.

Table S5. Primary global geographical-background tests across all vitamins.

Vitamin	*N*	*F*	df	Partial η^2	*p*	BH-FDR *q*
**Vitamin A (total RE)**	109	1.953	2, 105	0.036	0.147	0.202
**Vitamin D**	109	2.626	2, 105	0.048	0.077	0.121
**Vitamin E**	109	4.828	2, 105	0.084	0.010	0.034
**Vitamin K1 (phylloquinone)**	109	4.575	2, 105	0.080	0.012	0.034
**Thiamin (B1)**	109	0.981	2, 105	0.018	0.378	0.416
**Riboflavin (B2)**	109	3.148	2, 105	0.057	0.047	0.086
**Niacin (B3, total NE)**	109	5.023	2, 105	0.087	0.008	0.034
**Vitamin B6**	109	5.594	2, 105	0.096	0.005	0.034
**Folate (B9, total DFE)**	109	1.400	2, 105	0.026	0.251	0.307
**Vitamin B12**	109	3.952	2, 105	0.070	0.022	0.049
**Vitamin C**	109	0.425	2, 105	0.008	0.655	0.655

Primary models compared Africa, Asia and Europe and were adjusted for total energy intake. Multiple testing was controlled using the Benjamini–Hochberg false-discovery rate.

Table S6. Primary pairwise geographical-background comparisons across all vitamins.

Vitamin	Contrast	*N*	Adjusted difference (SD)	95% CI	*p*	BH-FDR *q*
**Vitamin A (total RE)**	Asia - Africa	109	0.025	-0.311 to 0.360	0.885	0.912
	Europe - Africa	109	0.341	-0.027 to 0.709	0.069	0.176
	Asia - Europe	109	-0.316	-0.691 to 0.058	0.097	0.228
**Vitamin D**	Asia - Africa	109	-0.423	-0.798 to -0.048	0.028	0.091
	Europe - Africa	109	-0.109	-0.521 to 0.303	0.602	0.764
	Asia - Europe	109	-0.314	-0.733 to 0.104	0.140	0.285
**Vitamin E**	Asia - Africa	109	0.359	0.102 to 0.616	0.007	0.044
	Europe - Africa	109	0.354	0.071 to 0.637	0.015	0.053
	Asia - Europe	109	0.005	-0.282 to 0.292	0.973	0.973
**Vitamin K1 (phylloquinone)**	Asia - Africa	109	0.386	0.020 to 0.752	0.039	0.112
	Europe - Africa	109	0.583	0.182 to 0.985	0.005	0.044
	Asia - Europe	109	-0.197	-0.605 to 0.211	0.340	0.535
**Thiamin (B1)**	Asia - Africa	109	-0.061	-0.246 to 0.125	0.519	0.684
	Europe - Africa	109	0.086	-0.118 to 0.289	0.407	0.583
	Asia - Europe	109	-0.146	-0.353 to 0.061	0.164	0.301
**Riboflavin (B2)**	Asia - Africa	109	-0.158	-0.422 to 0.105	0.235	0.388
	Europe - Africa	109	0.213	-0.076 to 0.502	0.147	0.285

Vitamin	Contrast	*N*	Adjusted difference (SD)	95% CI	*p*	BH-FDR *q*
	Asia - Europe	109	-0.372	-0.665 to -0.078	0.014	0.053
**Niacin (B3, total NE)**	Asia - Africa	109	-0.319	-0.558 to -0.081	0.009	0.044
	Europe - Africa	109	0.053	-0.209 to 0.315	0.691	0.787
	Asia - Europe	109	-0.372	-0.638 to -0.106	0.007	0.044
**Vitamin B6**	Asia - Africa	109	-0.355	-0.619 to -0.090	0.009	0.044
	Europe - Africa	109	0.098	-0.193 to 0.389	0.505	0.684
	Asia - Europe	109	-0.453	-0.748 to -0.157	0.003	0.044
**Folate (B9, total DFE)**	Asia - Africa	109	-0.044	-0.330 to 0.242	0.762	0.811
	Europe - Africa	109	0.213	-0.101 to 0.528	0.181	0.314
	Asia - Europe	109	-0.257	-0.577 to 0.062	0.113	0.249
**Vitamin B12**	Asia - Africa	109	-0.413	-0.722 to -0.104	0.009	0.044
	Europe - Africa	109	-0.052	-0.392 to 0.287	0.760	0.811
	Asia - Europe	109	-0.361	-0.706 to -0.015	0.041	0.112
**Vitamin C**	Asia - Africa	109	-0.155	-0.487 to 0.178	0.359	0.538
	Europe - Africa	109	-0.079	-0.445 to 0.287	0.669	0.787
	Asia - Europe	109	-0.076	-0.447 to 0.296	0.687	0.787

Pairwise contrasts were obtained from the primary energy-adjusted geographical-background models. Multiple testing was controlled using the Benjamini–Hochberg false-discovery rate.

Table S7. Fully adjusted HC3 sensitivity analysis for sex comparisons.

Vitamin	Contrast	*N*	Adjusted difference (SD)	HC3 95% CI	*p*	BH-FDR *q*
**Vitamin A (total RE)**	Male - Female	111	-0.526	-0.849 to -0.202	0.002	0.019
**Vitamin D**	Male - Female	111	-0.026	-0.440 to 0.388	0.903	0.978
**Vitamin E**	Male - Female	111	-0.296	-0.541 to -0.051	0.019	0.093
**Vitamin K1 (phylloquinone)**	Male - Female	111	-0.535	-1.003 to -0.067	0.025	0.093
**Thiamin (B1)**	Male - Female	111	0.106	-0.075 to 0.287	0.250	0.457
**Riboflavin (B2)**	Male - Female	111	-0.083	-0.373 to 0.206	0.570	0.896
**Niacin (B3, total NE)**	Male - Female	111	0.199	-0.057 to 0.456	0.127	0.279
**Vitamin B6**	Male - Female	111	0.294	-0.049 to 0.636	0.092	0.252
**Folate (B9, total DFE)**	Male - Female	111	-0.010	-0.309 to 0.289	0.946	0.978
**Vitamin B12**	Male - Female	111	0.006	-0.407 to 0.418	0.978	0.978
**Vitamin C**	Male - Female	111	-0.026	-0.399 to 0.348	0.892	0.978

Sensitivity models included age, geographical background and total energy intake and used HC3 robust covariance estimation.

Table S8. Fully adjusted HC3 sensitivity analysis for global geographical-background tests.

Vitamin	*N*	HC3 F	df	Partial η^2	*p*	BH-FDR *q*
**Vitamin A (total RE)**	109	2.048	2, 103	0.044	0.134	0.184
**Vitamin D**	109	3.110	2, 103	0.064	0.049	0.077
**Vitamin E**	109	4.651	2, 103	0.093	0.012	0.037
**Vitamin K1 (phylloquinone)**	109	4.509	2, 103	0.096	0.013	0.037
**Thiamin (B1)**	109	1.137	2, 103	0.021	0.325	0.357
**Riboflavin (B2)**	109	3.765	2, 103	0.073	0.026	0.058
**Niacin (B3, total NE)**	109	4.505	2, 103	0.094	0.013	0.037
**Vitamin B6**	109	6.027	2, 103	0.090	0.003	0.037
**Folate (B9, total DFE)**	109	1.845	2, 103	0.032	0.163	0.200
**Vitamin B12**	109	3.336	2, 103	0.075	0.039	0.072
**Vitamin C**	109	0.343	2, 103	0.007	0.711	0.711

Sensitivity models included age, sex and total energy intake and used HC3 robust covariance estimation.

Table S9. Fully adjusted HC3 sensitivity analysis for pairwise geographical-background comparisons.

Vitamin	Contrast	*N*	Adjusted difference (SD)	HC3 95% CI	*p*	BH-FDR *q*
**Vitamin A (total RE)**	Asia - Africa	109	0.040	-0.286 to 0.366	0.808	0.840
	Europe - Africa	109	0.374	-0.018 to 0.767	0.061	0.156
	Asia - Europe	109	-0.334	-0.710 to 0.041	0.080	0.166
**Vitamin D**	Asia - Africa	109	-0.470	-0.852 to -0.088	0.016	0.054
	Europe - Africa	109	-0.049	-0.459 to 0.361	0.814	0.840
	Asia - Europe	109	-0.422	-0.893 to 0.049	0.079	0.166
**Vitamin E**	Asia - Africa	109	0.352	0.078 to 0.626	0.012	0.054
	Europe - Africa	109	0.388	0.113 to 0.663	0.006	0.040
	Asia - Europe	109	-0.036	-0.298 to 0.226	0.785	0.840
**Vitamin K1 (phylloquinone)**	Asia - Africa	109	0.395	-0.009 to 0.799	0.055	0.152
	Europe - Africa	109	0.624	0.212 to 1.036	0.003	0.040
	Asia - Europe	109	-0.229	-0.566 to 0.108	0.181	0.298
**Thiamin (B1)**	Asia - Africa	109	-0.072	-0.257 to 0.113	0.440	0.626
	Europe - Africa	109	0.089	-0.146 to 0.323	0.455	0.626
	Asia - Europe	109	-0.161	-0.377 to 0.055	0.143	0.263
**Riboflavin (B2)**	Asia - Africa	109	-0.182	-0.466 to 0.101	0.205	0.323
	Europe - Africa	109	0.250	-0.052 to 0.551	0.104	0.201

Vitamin	Contrast	*N*	Adjusted difference (SD)	HC3 95% CI	*p*	BH-FDR *q*
	Asia - Europe	109	-0.432	-0.744 to -0.120	0.007	0.040
**Niacin (B3, total NE)**	Asia - Africa	109	-0.336	-0.579 to -0.093	0.007	0.040
	Europe - Africa	109	0.055	-0.221 to 0.331	0.692	0.827
	Asia - Europe	109	-0.391	-0.703 to -0.079	0.015	0.054
**Vitamin B6**	Asia - Africa	109	-0.355	-0.607 to -0.103	0.006	0.040
	Europe - Africa	109	0.076	-0.254 to 0.406	0.649	0.827
	Asia - Europe	109	-0.431	-0.735 to -0.127	0.006	0.040
**Folate (B9, total DFE)**	Asia - Africa	109	-0.061	-0.368 to 0.245	0.693	0.827
	Europe - Africa	109	0.233	-0.089 to 0.554	0.154	0.268
	Asia - Europe	109	-0.294	-0.609 to 0.022	0.068	0.159
**Vitamin B12**	Asia - Africa	109	-0.425	-0.768 to -0.083	0.015	0.054
	Europe - Africa	109	-0.031	-0.331 to 0.270	0.840	0.840
	Asia - Europe	109	-0.395	-0.756 to -0.034	0.032	0.097
**Vitamin C**	Asia - Africa	109	-0.150	-0.510 to 0.210	0.410	0.615
	Europe - Africa	109	-0.079	-0.497 to 0.339	0.709	0.827
	Asia - Europe	109	-0.071	-0.475 to 0.332	0.727	0.827

Pairwise sensitivity contrasts were obtained from models including age, sex and total energy intake with HC3 robust covariance estimation.

Table S10. Full dietary-reference comparison table.

Vitamin	Reference benchmark	Reference type	Reference value / rule	Below benchmark, *n*/*N*	Below benchmark, % (95% CI)
**Vitamin A (total RE)**	UK LRNI	LRNI	Men 300 μg RE/day; women 250 μg RE/day	5/111	4.5 (1.9–10.1)
	UK RNI	RNI	Men 700 μg RE/day; women 600 μg RE/day	38/111	34.2 (26.1–43.5)
**Thiamin (B1)**	UK LRNI	LRNI	0.23 mg/1000 kcal × participant energy	0/111	0.0 (0.0–3.3)
	UK RNI	RNI	0.40 mg/1000 kcal × participant energy	0/111	0.0 (0.0–3.3)
**Riboflavin (B2)**	UK LRNI	LRNI	0.8 mg/day	1/111	0.9 (0.2–4.9)
	UK RNI	RNI	Men 1.3 mg/day; women 1.1 mg/day	8/111	7.2 (3.7–13.6)
**Niacin (B3, total NE)**	UK LRNI	LRNI	4.4 mg NE/1000 kcal × participant energy	0/111	0.0 (0.0–3.3)
	UK RNI	RNI	6.6 mg NE/1000 kcal × participant energy	0/111	0.0 (0.0–3.3)
**Vitamin B6**	UK LRNI	LRNI	11 μg/g dietary protein × participant protein	0/111	0.0 (0.0–3.3)
	UK RNI	RNI	15 μg/g dietary protein × participant protein	1/111	0.9 (0.2–4.9)
**Vitamin B12**	UK LRNI	LRNI	1.0 μg/day	1/111	0.9 (0.2–4.9)
	UK RNI	RNI	1.5 μg/day	2/111	1.8 (0.5–6.3)
**Vitamin C**	UK LRNI	LRNI	10 mg/day	0/111	0.0 (0.0–3.3)
	UK RNI	RNI	40 mg/day	6/111	5.4 (2.5–11.3)
**Vitamin D**	UK RNI	RNI	10 μg/day	105/111	94.6 (88.7–97.5)
**Folate (B9, total DFE)**	EFSA AR	AR	250 μg DFE/day	31/111	27.9 (20.4–36.9)

Vitamin	Reference benchmark	Reference type	Reference value / rule	Below benchmark, *n*/*N*	Below benchmark, % (95% CI)
	EFSA PRI	PRI	330 µg DFE/day	54/111	48.6 (39.6–57.8)
**Vitamin E**	EFSA AI	AI	α-tocopherol: men 13 mg/day; women 11 mg/day	NOT CLASSIFIED	—
**Vitamin K1 (phylloquinone)**	UK safe intake	safe intake	~1 µg/kg body weight/day × participant weight	44/111	39.6 (31.0–48.9)
	EFSA AI	AI	70 µg/day	47/111	42.3 (33.6–51.6)

LRNI, RNI, AR, PRI, AI and safe-intake values have different interpretive meanings. Comparisons with AI or safe-intake values are descriptive and should not be interpreted as estimates of individual inadequacy. Vitamin E remains not classified against the EFSA AI.

Table S11. Agreement between raw CoFID-reconstructed vitamin totals and original Nutritics totals at participant level.

Vitamin	*N*	Pearson *r*	Spearman ρ	Median absolute relative difference (%)	Composition coverage (%)
**Folate (CoFID total)**	111	0.935	0.897	4.4 [2.0–15.2]	97.5
**Niacin (B3, total NE)**	111	0.988	0.975	4.3 [1.6–7.4]	97.5
**Riboflavin (B2)**	111	0.993	0.980	6.4 [3.7–10.2]	97.8
**Thiamin (B1)**	111	0.990	0.980	3.8 [2.0–7.4]	97.9
**Vitamin A (total RE)**	111	0.965	0.941	12.9 [7.1–22.6]	92.9
**Vitamin B12**	111	0.977	0.949	8.8 [2.9–14.8]	98.0
**Vitamin B6**	111	0.826	0.842	9.6 [3.5–19.5]	97.3
**Vitamin C**	111	0.993	0.987	4.7 [2.3–9.0]	95.4
**Vitamin D**	111	0.972	0.977	5.3 [2.0–16.7]	96.3
**Vitamin E**	111	0.944	0.965	5.5 [2.6–9.5]	88.4
**Vitamin K1 (phylloquinone)**	111	0.974	0.956	23.2 [16.2–34.3]	47.7

For folate, the comparison involves CoFID total folate (FOLT) and the original Nutritics natural-folate variable. As these variables are not compositionally identical, the reported correlation and relative differences should be interpreted descriptively rather than as evidence of agreement between equivalent folate measures. Composition coverage is the percentage of cohort food grams with usable CoFID composition for the vitamin.

Table S12. Concordance of food-group source structure before and after calibration.

Vitamin	Pearson *r*	Spearman *ρ*	Top-five overlap
**Folate (CoFID total)**	0.997	0.998	5/5
**Niacin (B3, total NE)**	1.000	0.998	5/5
**Riboflavin (B2)**	1.000	1.000	5/5
**Thiamin (B1)**	1.000	1.000	5/5
**Vitamin A (total RE)**	0.999	0.998	5/5
**Vitamin B12**	1.000	1.000	5/5
**Vitamin B6**	1.000	0.998	5/5
**Vitamin C**	1.000	1.000	5/5
**Vitamin D**	0.999	0.998	5/5
**Vitamin E**	0.999	0.998	5/5
**Vitamin K1 (phylloquinone)**	1.000	1.000	5/5

Pearson and Spearman correlations compare food-group percentage profiles before and after calibration. Top-five overlap is the number of food groups shared between the two top-five rankings.

Table S13. Exploratory top-five food-group contributors to vitamin K1.

Food group	Raw CoFID contribution (%)	Calibrated contribution (%)	Rank	Top-five cumulative (%)
**Vegetables**	89.9	88.8	1	98.2
**Fruit**	4.9	5.6	2	98.2
**Dairy/Alternatives**	1.5	1.7	3	98.2
**Cereals/Grains**	1.2	1.2	4	98.2
**Red/processed meat**	0.8	0.9	5	98.2

Vitamin K1 food-source attribution is exploratory because usable CoFID composition covered approximately 47.7% of cohort food grams. Negative derived oil-swap K1 values were treated as unavailable rather than zero before K1-only recalibration.
